# Effect of physical activity on incident atrial fibrillation in individuals with varying duration of diabetes: a nationwide population study

**DOI:** 10.1186/s12933-024-02194-2

**Published:** 2024-03-30

**Authors:** JungMin Choi, So‑Ryoung Lee, Eue-Keun Choi, Kyung-Yeon Lee, Hyo-Jeong Ahn, Soonil Kwon, Kyung‑Do Han, Seil Oh, Gregory Y. H. Lip

**Affiliations:** 1 Division of cardiology, Department of Internal Medicine, Seoul National University Hospital, Seoul, Republic of Korea; 2 Department of Internal Medicine, Seoul National University College of Medicine and Seoul National University Hospital, 101 Daehak-ro, Jongno‐gu, Seoul, 03080 Republic of Korea; 3 Statistics and Actuarial Science, Soongsil University, Seoul, Republic of Korea; 4 Liverpool Center for Cardiovascular Science, University of Liverpool, Liverpool John Moores University and Liverpool Chest & Heart Hospital, Liverpool, UK; 5 Danish Center for Health Services Research, Department of Clinical Medicine, Aalborg University, Aalborg, Denmark

**Keywords:** Atrial fibrillation, Type 2 diabetes mellitus, Physical activity

## Abstract

**Background:**

Diabetes mellitus (DM) duration affects incident atrial fibrillation (AF) risk; the effect of physical activity on mitigating AF risk related to varying DM duration remains unknown. We assessed the effect of physical activity on incident AF in patients with DM with respect to known DM duration.

**Methods:**

Patients with type 2 DM who underwent the Korean National Health Insurance Service health examination in 2015–2016 were grouped by DM duration: new onset and < 5, 5–9, and ≥ 10 years. Physical activity was classified into four levels: 0, < 500, 500–999, 1,000–1,499, and ≥ 1,500 metabolic equivalent task (MET)-min/week, with the primary outcome being new-onset AF.

**Results:**

The study enrolled 2,392,486 patients (aged 59.3 ± 12.0 years, 39.8% female) with an average follow-up of 3.9 ± 0.8 years and mean DM duration of 5.3 ± 5.1 years. Greater physical activity was associated with a lower AF risk. Lowering of incident AF risk varied with different amounts of physical activity in relation to known DM duration. Among patients with new-onset DM, DM duration < 5 years and 5–9 years and 1,000–1,499 MET-min/week exhibited the lowest AF risk. Physical activity ≥ 1,500 MET-min/week was associated with the lowest incident AF risk in patients with DM duration ≥ 10 years (by 15%), followed DM duration of 5–9 years (12%) and < 5 years (9%) (*p*-for-interaction = 0.002).

**Conclusions:**

Longer DM duration was associated with a high risk of incident AF, while increased physical activity generally reduced AF risk. Engaging in > 1,500 MET-min/week was associated with the greatest AF risk reduction in patients with longer DM duration, highlighting the potential benefits of higher activity levels for AF prevention.

**Supplementary Information:**

The online version contains supplementary material available at 10.1186/s12933-024-02194-2.

## Background

Type 2 diabetes mellitus (T2DM) increases the susceptibility to cardiovascular diseases such as myocardial infarction (MI), heart failure, and atrial fibrillation (AF) [1]. In line with recent guidelines on diabetes mellitus (DM), management recommendations now encompass pharmacological approaches with holistic lifestyle modifications [2]. This includes exercise, which is vital for reducing cardiovascular morbidity and mortality regardless of the specific type [3, 4].

In individuals with T2DM, AF substantially affects morbidity and mortality outcomes [5]. Consequently, considerable attention has been devoted to AF prevention, focusing on identifying risk factors, including lifestyle factors. The established risk factors for AF include advanced age and comorbidities such as hypertension and DM [6, 7]. Notably, unhealthy lifestyle habits such as obesity, smoking, and alcohol consumption have been linked to an increased incidence of AF [8, 9].

The relationship between exercise and AF in the general population exhibits a complex and nonlinear pattern [10, 11]. In individuals with T2DM, recent studies have highlighted the beneficial effects of physical activity on AF [9, 12]. However, the influence of the amount of physical activity on mitigating AF risk, related to varying durations of DM duration remains unknown.

This study aimed to assess the effect of physical activity on incident AF in patients with DM with respect to known DM duration, using a large-scale nationwide population-based cohort.

## Methods

Data were extracted from the extensive nationwide claims database of the Korean National Health Insurance Service (NHIS), which provides coverage for the entire South Korean population. The NHIS database contains a wide range of information, including demographic variables; mortality data; medical expenses; diagnoses classified by the International Classification of Diseases, Tenth Revision, Clinical Modification (ICD-10-CM); utilization of inpatient and outpatient services; and prescription records [13]. Additionally, the National Health Screening Program for chronic diseases caters to individuals over 19 years of age and encompasses data from physical examinations, laboratory results, and self-reported questionnaires [14].

### Study population

**Fig. 1:**
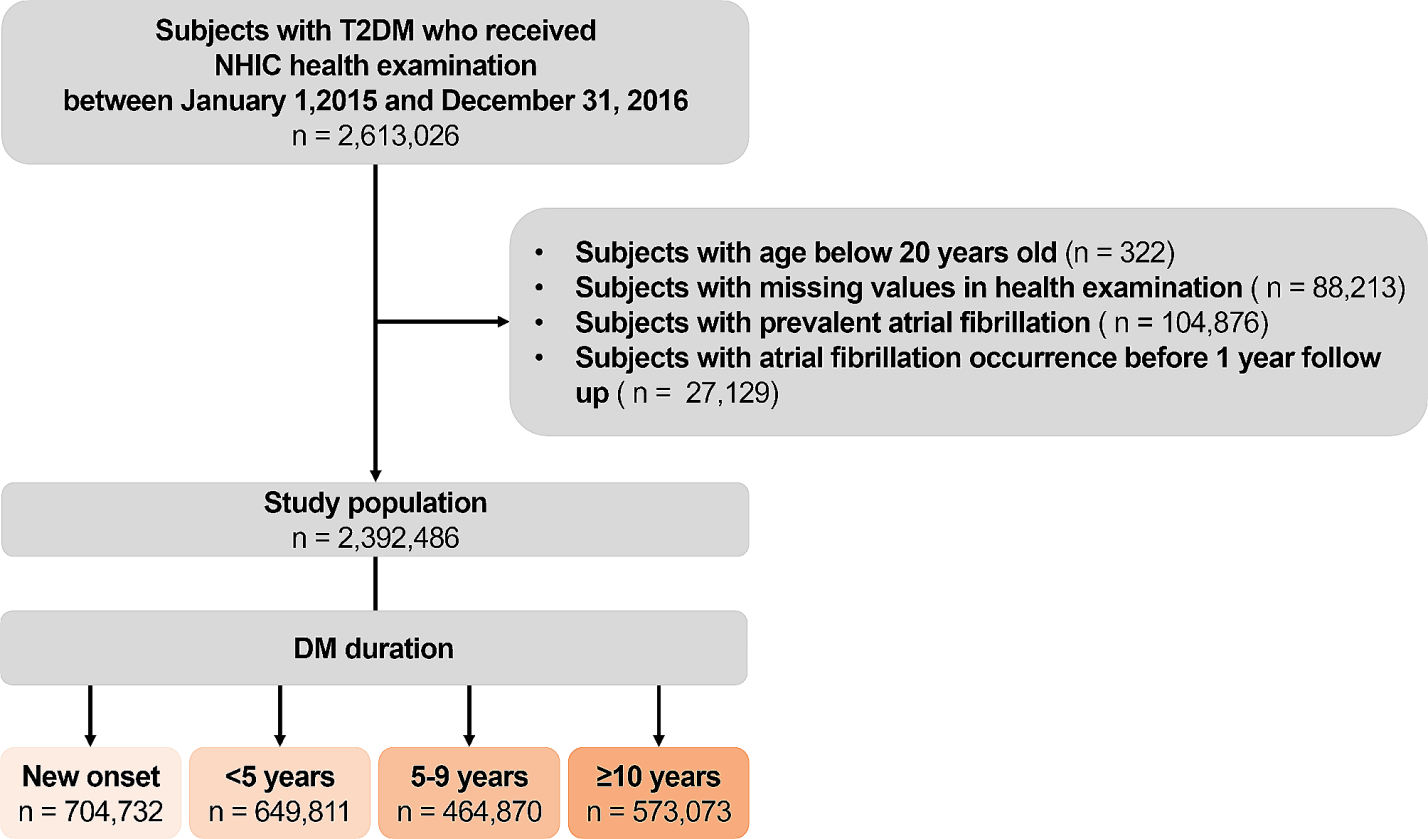
Study flow. *Abbreviation* T2DM, type 2 diabetes mellitus; NHIC, national health insurance cooperation

Figure 1 provides an overview of the patient selection process. The initial pool consisted of patients with DM who underwent a National Health Insurance Corporation health examination between January 1, 2015, and December 31, 2016, resulting in a total of 2,613,026 individuals. Patients aged < 20 years (*n* = 322), those with missing data (*n* = 88,213), those with prevalent AF before enrollment (*n* = 104,876), and those who developed AF before 1-year follow-up (*n* = 27,129) were excluded. Subsequently, the remaining participants were categorized into four groups based on the duration of their DM: new-onset (first diagnosed at baseline health examination), < 5 years, 5–9 years, and ≥ 10 years. This categorization of DM duration has been widely used since study by Zoungas and his colleagues and have shown to be associated with elevated cardiovascular disease risk [15–17]. 

### Measurement of the amount of physical activity

The intensity and frequency of exercise in health examinations were collected via self-report structured questionnaires using a 7-d recall method. The survey questionnaire used to assess exercise was adapted from the International Physical Activity Questionnaire endorsed by the World Health Organization. This survey consisted of three questions inquiring about the frequency of: (1) light-intensity physical activity (e.g., slow or leisurely walking) for at least 30 min, (2) moderate-intensity physical activity (e.g., brisk walking, slow cycling, or tennis doubles) for at least 30 min, and (3) vigorous-intensity physical activity (e.g., running, cycling over 15 km/h, climbing, or participating in an aerobics class) for at least 20 min during the past week. Previous studies confirmed the validity and reliability of this survey method [18, 19]. Regular physical activity was defined as performing moderate-intensity physical activity for more than 30 min at least five times per week or strenuous physical activity for more than 20 min at least thrice per week (***regular physical activity 1***) [20]. The criterion for the presence of physical activity involved engaging in either moderate physical activity for a minimum of 30 min once per week or strenuous physical activity for more than 20 min at least once per week (referred to as ***regular physical activity 2***).

To determine physical activity-related energy expenditure (metabolic equivalent task [MET]-min/week), we assigned pre-specified values to each intensity of activity (2.9 for light-intensity, 4.0 for moderate-intensity, and 7.0 for vigorous-intensity activities). Subsequently, the sum of the products of frequency, intensity, and duration was calculated for each activity. To assess the relationship between physical activity and the risk of AF, an analysis was conducted on the hazard ratios (HRs) for incident AF, categorized based on energy expenditure using the previously mentioned method [12, 21, 22]. Energy expenditure was divided into four groups: <500, 500 to 999, 1000 to 1499, and ≥ 1500 MET-min/week, based on the level of energy expenditure [22].

### Covariates

Detailed definitions of the inclusion and exclusion criteria (AF, DM), as well as the specified comorbidities (hypertension, dyslipidemia, chronic kidney disease [CKD], proteinuria, heart failure, prior MI, prior stroke, and peripheral artery disease [PAD]), health behaviors (smoking, alcohol consumption, and exercise), and household income are provided in Supplementary Table S1. The administration of oral antihyperglycemic medications, including sulfonylureas, metformin, meglitinides, thiazolidinediones, dipeptidyl peptidase-4 inhibitors, α-glucosidase inhibitors, and insulin were evaluated. General health examinations included measurements of systolic and diastolic blood pressure, BMI, and waist circumference. Additionally, laboratory analyses included estimated glomerular filtration rate (eGFR) and fasting glucose, total cholesterol, triglyceride, HDL cholesterol, and LDL cholesterol levels [23–25]. A self-reported questionnaire was used to gather data on smoking habits (never, ex-, or current) and alcohol consumption patterns (none, mild to moderate, or heavy) [8, 9, 23].

### Study outcomes and follow-up

The primary outcome assessed throughout the follow-up period was the incidence of AF. AF was defined as the initial diagnosis of related ICD-10-CM codes (I48; AF and atrial flutter) during at least two separate outpatient clinic visits or one hospital admission, or at death [23]. Patients were monitored from the index date until the occurrence of incident AF, disqualification from the NHIS due to immigration, death, or the conclusion of the study (December 31, 2020), whichever occurred first.

### Statistical analysis

Numerical data are presented as the mean ± standard deviation for variables with a normal distribution and as the geometric mean with interquartile range for those without a normal distribution. Categorical variables were presented as numbers and frequencies. Baseline characteristics were compared using one-way analysis of variance to assess differences in continuous variables, whereas chi-square tests were conducted to evaluate group differences in categorical variables. The incidence rate (IR) of AF events was determined as the number of new AF events per 1,000 person-years. Kaplan–Meier survival curves of cumulative AF incidence were compared using the log-rank test. Cox proportional hazards regression models were used to calculate HRs and the corresponding 95% CIs. A stepwise approach was employed involving five Cox models with varying covariate adjustments: (i) unadjusted model (model 1); (ii) model adjusted for age and sex (model 2); (iii) model adjusted for age, sex, comorbidities (hypertension, dyslipidemia, CKD), antihyperglycemic agent use (more than three drug prescriptions, insulin), BMI, fasting glucose, low income, and social behavior (smoking, alcohol consumption) (model 3); (iv) model 3 with additional comorbidities (heart failure, prior MI, prior stroke, and PAD) (model 4); and (v) model 4 with the addition of eGFR and proteinuria (model 5). To evaluate the effect of covariates on AF risk across the different DM duration, we performed a univariable and a multivariable logistic regression for the covariates used in Model 5.

Statistical significance was set at *p* < 0.05. All statistical analyses were performed using SAS version 9.4 (SAS Institute, Cary, North Carolina, USA).

## Results

### Baseline characteristics

The final study population consisted of 2,392,486 individuals. Based on DM duration, the participants were divided into four groups: new-onset (*n* = 707,432), < 5 years (*n* = 649,811), 5–9 years (*n* = 464,870), and ≥ 10 years (*n* = 573,073). Table 1 presents the baseline characteristics of the study population according to DM duration.

**Table 1:** Baseline characteristics of the study population

	Total (*n* = 2,392,486)	DM duration				*p*-value
		New onset (*n* = 704,732)	< 5 years (*n* = 649,811)	5–9 years (*n* = 464,870)	≥ 10 years (*n* = 573,073)	
**Age, years**						
Mean ± SD	59.3 ± 12.0	53.5 ± 12.3	58.5 ± 11.3	61.8 ± 10.5	65.2 ± 9.8	< 0.001
< 40	5.0	12.0	4.1	1.4	0.5	
40–64	61.7	70.3	67.0	59.7	46.8	
≥ 65	33.2	17.6	28.9	38.9	52.7	
Sex (women)	39.8	30.5	42.7	43.4	44.9	< 0.001
**Comorbidities**						
Hypertension	57.9	43.9	58.7	64.9	68.5	< 0.001
Dyslipidemia	56.4	35.3	65.7	65.1	64.6	< 0.001
CKD	9.4	4.5	6.5	10.2	18.2	< 0.001
Proteinuria	6.9	5.3	5.5	6.5	10.8	< 0.001
Heart failure	3.0	1.4	3.2	3.4	4.5	< 0.001
Prior MI	5.2	2.7	4.6	6.2	8.1	< 0.001
Prior stroke	12.2	5.6	10.2	14.5	20.5	< 0.001
PAD	17.6	7.8	16.8	21.7	27.0	< 0.001
**Social history**						
Smoking						< 0.001
Non-smoker	54.8	46.5	55.5	57.8	61.8	
Ex-smoker	22.3	22.6	22.0	22.3	22.2	
Current smoker	22.9	30.9	22.4	19.9	16.0	
Alcohol consumption						< 0.001
Non-drinker	57.3	42.3	59.4	62.9	69.0	
Mild to moderate (0–30 g/day)	33.5	44.3	31.9	29.5	25.1	
Heavy (≥ 30 g/day)	9.2	13.4	8.7	7.7	5.9	
Regular physical activity 1^a^	21.7	20.4	20.9	22.3	23.7	< 0.001
Regular physical activity 2^b^	50.3	54.7	49.7	48.8	46.9	< 0.001
METs-min/week	643.0 ± 638.0	637.4 ± 612.0	626.8 ± 626.7	646.5 ± 647.3	665.3 ± 672.9	< 0.001
Low income	21.6	20.7	22.0	22.8	21.2	< 0.001
**Medication**						
OHA ≥ 3	24.3	0.0	18.4	35.3	51.8	< 0.001
Insulin usage	8.4	0.0	6.8	8.3	20.6	< 0.001
DM duration (years)	5.3 ± 5.1	-	2.0 ± 1.6	7.4 ± 1.5	12.7 ± 1.3	< 0.001
**Health examination**						
BMI (kg/m^2^)	25.3 ± 3.5	25.6 ± 3.7	25.8 ± 3.6	25.3 ± 3.4	24.6 ± 3.2	< 0.001
Obesity	51.1	54.4	56.3	50.7	41.6	< 0.001
WC (cm)	86.1 ± 9.0	86.1 ± 9.2	86.8 ± 9.1	86.2 ± 8.8	85.3 ± 8.6	< 0.001
Abdominal Obesity	40.9	38.9	44.6	42.2	38.2	< 0.001
SBP (mmHg)	128.5 ± 15.0	129.5 ± 15.3	127.7 ± 14.6	128.0 ± 14.8	128.6 ± 15.3	< 0.001
DBP (mmHg)	78.1 ± 9.9	80.3 ± 10.3	78.4 ± 9.7	77.3 ± 9.4	75.8 ± 9.6	< 0.001
**Laboratory results**						
eGFR (mL/min/1.73 m2)	89.6 ± 53.0	93.7 ± 60.1	91.8 ± 51.4	88.6 ± 49.7	82.8 ± 47.3	< 0.001
Fasting Glucose (mg/dL)	145.0 ± 45.9	151.7 ± 39.3	138.2 ± 46.5	140.7 ± 45.3	147.7 ± 51.4	< 0.001
Total cholesterol (mg/dL)	185.9 ± 43.7	207.5 ± 41.9	184.7 ± 43.3	174.7 ± 39.3	169.8 ± 38.8	< 0.001
HDL-C (mg/dL)	51.0 ± 14.7	52.6 ± 16.3	50.7 ± 14.1	50.4 ± 13.7	49.9 ± 14.0	< 0.001
LDL-C (mg/dL)	103.6 ± 38.5	119.8 ± 38.3	102.6 ± 38.4	94.9 ± 35.0	92.0 ± 34.3	< 0.001
TG (mg/dL)^c^	138.3(138.2–138.4)	156.7(156.5–157.0)	139.6 (139.4–139.8)	130.6 (130.4–130.8)	122.9 (122.7–123.1)	< 0.001

Categorical variables were presented as a percentage and continuous variables were presented as mean and standard deviation

*Abbreviation* CKD, chronic kidney disease; DBP, diastolic blood pressure; DM, diabetes mellitus; eGFR, estimated glomerular filtration rate; MI, myocardial infarction; OHA, oral antihyperglycemic agents; PAD, peripheral artery disease; SBP, systolic blood pressure; TG, triglyceride; WC, waist circumference

^a^Performing a moderate physical activity more than 30 min at least 5 times per week or strenuous physical activity more than 20 min at least 3 times per week

^b^Performing a moderate physical activity more than 30 min at least 1 time per week or strenuous physical activity more than 20 min at least 1 time per week

^c^TG was presented as geometric mean (95% confidence interval)

The study cohort had a mean age of 59.3 ± 12.0 years and was 39.8% female. The most prevalent comorbidity was hypertension (57.9%), followed by dyslipidemia (56.4%). As the duration of diabetes increased, there was a steady and linear increase in the proportion of comorbidities, such as hypertension, CKD, heart failure, prior MI, prior stroke, and PAD. The mean DM duration was 5.3 ± 5.1 years, and as the duration increased, there was a consistent linear rise in the prescription of more than three oral antihyperglycemic agents (0.0% in the new-onset group, 18.4% in the < 5 years group, 35.3% in the 5–9 years group, and 51.8% in the ≥ 10 years group) and insulin (0.0% in the new onset group, 6.8% in the < 5 years group, 8.3% in the 5–9 years group, and 20.6% in the ≥ 10 years group). The percentage of current smokers and alcohol consumers was highest among individuals in the new-onset DM duration group and lowest among those with a DM duration ≥ 10 years (30.9% vs. 16.0% for current smokers; 57.3% vs. 31% for alcohol consumers). The previously known cardiovascular risk factors mostly demonstrated a consistent effect on AF risk despite the duration of DM (Supplementary Table S2 and S3). However, there was variation with the size of effect with different DM duration.

### Physical activity according to the duration of diabetes

In the total study population, the proportion of patients performing regular ***physical activity 2*** decreased with an increasing duration of DM (Table 1). Conversely, the proportion of those performing regular ***physical activity 1*** increased as the DM duration increased (Table 1). The mean physical activity level of the total population was 643.0 ± 638.0 MET-min/week. Within each subgroup based on varying DM duration, the mean physical activity increased with an increase in DM duration (Table 1).

Supplementary Table S4 provides a comparison of baseline characteristics based on different levels of physical activity and DM duration. In the new-onset DM group, those with a sedentary lifestyle were the oldest (mean age 55.5 years, *p* < 0.001), with the highest proportion of women (36.8%; *p* < 0.001) (Supplementary Table S4 (A)). The proportion of patients with dyslipidemia decreased with an increase in physical activity, but other comorbidities did not show a significant linear association.

### Physical activity and the risk of incident AF with different durations of diabetes

**Fig. 2:**
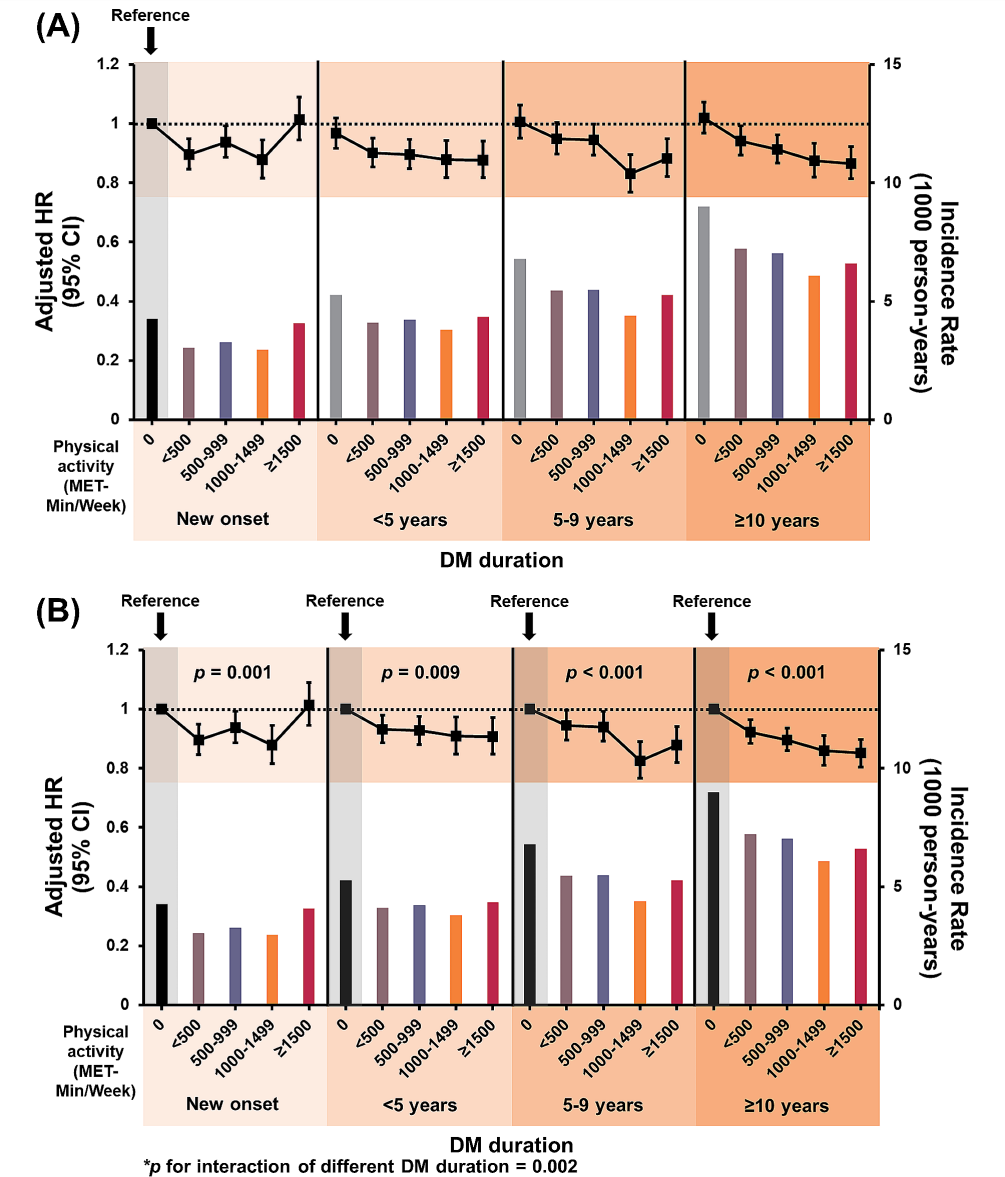
Adjusted hazard ratio and incidence rate of atrial fibrillation in subjects with different diabetes mellitus duration and physical activity (**A**) Reference as new onset with no physical activity (**B**) Reference as no physical activity in each DM duration group. *Abbreviation* CI, confidence interval; DM, diabetes mellitus; HR, hazard ratio

**Fig. 3:**
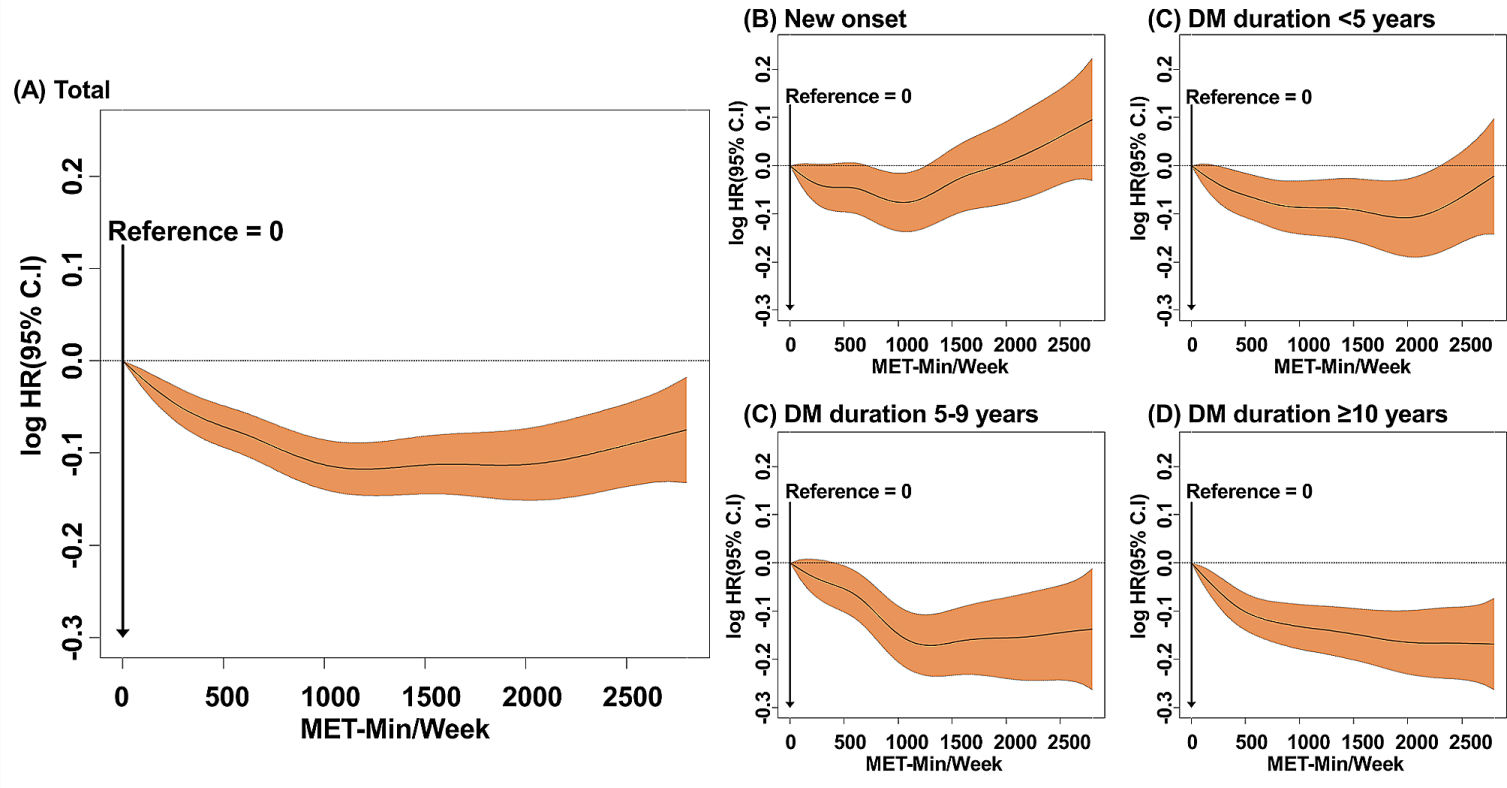
Cubic spline curve of incident AF in subjects with different diabetes duration and physical activity (**A**) Total (**B**) New onset (**C**) DM duration < 5 years (**D**) DM duration 5–10 years (**D**) DM duration. *Abbreviation* CI, confidence interval; DM, diabetes mellitus; HR, hazard ratio; Min, minute

Over a mean follow-up period of 3.9 ± 0.8 years, 46,674 patients (2.0% of total population; IR of 5.1 per 1,000 person-years) developed new-onset AF. In the total study population, all the ranges of physical activity were associated with a lower risk of AF compared to no physical activity (Table 2). The amount of physical activity associated with the lowest AF risk was 1,000–1,499 MET-min/week (adjusted HR 0.87; 95% CI 0.84–0.90) followed by ≥ 1,500 MET-min/week (adjusted HR 0.90, 95% CI 0.87–0.93) in the total study population (Table 2). The trends observed in this categorical analysis were consistent with those of the adjusted cubic spline curve (Figs. 2, 3A).

**Table 2:** Risk of incident AF according to DM duration and physical activity

DM Duration	METs-Min/week	N	AF	IR per 1000 PY	Model 5 Composite	*p*-value	Model 5 Subgroup	*p*-value	*p*-for-interaction
Total	0	544,927	12,904	6.20	1 (Reference)	< 0.001			
	< 500	650,353	11,858	4.74	0.93 (0.90–0.95)				
	500–999	648,499	12,104	4.84	0.92 (0.90–0.95)				
	1000–1499	284,775	4625	4.18	0.87 (0.84–0.90)				
	≥ 1500	263,932	5183	5.07	0.90 (0.87–0.93)				
New onset	0	151,684	2466	4.28	1 (Reference)	< 0.001	1 (Reference)	0.001	0.002
	< 500	199,056	2300	3.03	0.90 (0.85–0.95)		0.90 (0.85–0.95)		
	500–999	195,121	2440	3.28	0.94 (0.89–0.99)		0.94 (0.89–0.99)		
	1000–1499	88,335	1004	2.97	0.88 (0.82–0.95)		0.88 (0.82–0.95)		
	≥ 1500	70,536	1100	4.07	1.01 (0.95–1.09)		1.01 (0.95–1.09)		
< 5 years	0	150,864	3092	5.26	0.97 (0.916–1.02)		1 (Reference)	0.009	
	< 500	180,403	2897	4.11	0.90 (0.85–0.95)		0.93 (0.89–0.98)		
	500–999	175,075	2882	4.21	0.90 (0.85–0.95)		0.93 (0.88–0.98)		
	1000–1499	75,923	1135	3.80	0.88 (0.82–0.94)		0.91 (0.85–0.97)		
	≥ 1500	67,546	1146	4.33	0.88 (0.82–0.94)		0.91 (0.85–0.97)		
5–9 years	0	10,769	2814	6.78	1.01 (0.95–1.062		1 (Reference)	< 0.001	
	< 500	124,166	2632	5.46	0.95 (0.90–1.00)		0.95 (0.90–1.00)		
	500–999	125,148	2675	5.49	0.95 (0.89–1.00)		0.94 (0.89–0.99)		
	1000–1499	54,732	944	4.40	0.83 (0.77–0.90)		0.83 (0.77–0.89)		
	≥ 1500	53,132	1093	5.26	0.88 (0.82–0.95)		0.88 (0.82–0.94)		
≥ 10 years	0	134,687	4532	9.00	1.02 (0.97–1.07)		1 (Reference)	< 0.001	
	< 500	146,728	4029	7.22	0.94 (0.89–0.92)		0.92 (0.89–0.96)		
	500–999	153,155	4107	7.02	0.91 (0.87–0.96)		0.90 (0.86–0.94)		
	1000–1499	65,785	1542	6.08	0.88 (0.82–0.93)		0.86 (0.81–0.91)		
	≥ 1500	72,718	1844	6.60	0.87 (0.81–0.92)		0.85 (0.81–0.90)		

Model 5: adjusted for age, sex, comorbidities (hypertension, dyslipidemia, chronic kidney disease), anti-hyperglycemic agent usage (more than three drug prescription, insulin), BMI, fasting glucose, low income, social behavior (smoking, alcohol), heart failure, prior myocardial infarction, prior stroke, peripheral artery disease, estimated glomerular filtration rate and proteinuria

*Abbreviation* AF, atrial fibrillation; DM, diabetes mellitus; IR, incidence rate; PY, person-year

The crude IR and adjusted HR with model 5 of AF according to the amount of physical activity among the different DM duration groups are presented in Table 2; Fig. 2. Generally, in all groups with different DM durations, a greater amount of physical activity was associated with a lower risk of AF, although the specific amount of physical activity associated with the lowest risk varied among the DM duration groups (Table 2; Fig. 2). The adjusted HRs estimated using other models are presented in Supplementary Table S5.

In patients with new-onset AF, all ranges of physical activity exhibited a lower AF risk, except ≥ 1,500 MET-min/week. Notably, patients participating in physical activity within the range of 1,000 to 1,499 MET-min/week were the least susceptible to AF (adjusted HR 0.88, 95% CI 0.82–0.95) (Table 2; Fig. 2). The adjusted cubic spline curve further corroborated these trends, revealing a J-shaped correlation between the extent of physical activity and risk of AF (Fig. 3B).

Patients with a DM duration < 5 years, at each range of physical activity, displayed a lower AF risk than those with new-onset AF who did not engage in physical activity (Table 2; Fig. 2). Physical activity levels of 1,000 to 1,499 MET-min/week and ≥ 1,500 MET-min/week were both linked to a 9% lower AF risk compared to a DM duration < 5 years and no physical activity (Fig. 2B), and a 12% reduction in AF risk compared to new-onset AF with no physical activity (Fig. 2A).

Patients with a DM duration of 5–9 years, who engaged in physical activity at levels of 1,000 to 1,499 MET-min/week and ≥ 1,500 MET-min/week, demonstrated a significantly lower risk of AF by 17% and 12%, respectively, compared with those with new-onset AF who did not participate in physical activity (Table 2; Fig. 2A). These lower AF risks were also evident when the aforementioned patients were compared with those in the 5–9 years DM duration group who remained physically inactive (Fig. 2B).

Patients with a DM duration exceeding 10 years, across all degrees of physical activity, had a lower AF risk than those with new-onset AF who did not engage in physical activity (Table 2; Fig. 2A). In contrast to patients with a DM duration ≥ 10 years and no physical activity, those with greater levels of physical activity exhibited an enhanced reduction in the risk of new-onset AF; the risk reduction amounted to 8% for physical activity levels < 500 MET-min/week, 10% for physical activity levels of 500–999 MET-min/week, 14% for physical activity levels of 1,000–1,499 MET-min/week, and 15% for physical activity levels ≥ 1,500 MET-min/week (Fig. 2B).

Across all DM duration groups, the beneficial effect of a substantial amount of physical activity on the risk of AF was accentuated in patients with longer DM duration (p-for-interaction, *p* = 0.002) (Table 2; Fig. 2B). A J-shaped relationship between the magnitude of physical activity and AF risk was counteracted in patients with longer DM durations; specifically, those with DM durations of 10 years or more exhibited a diminished AF risk in tandem with escalated levels of exercise (Fig. 3D).

## Discussion

Our principal findings can be summarized as follows: (1) in patients with T2DM, all ranges of physical activity were associated with a lower AF risk compared to physical inactivity; (2) those engaging in physical activity at levels of 1000–1499 MET-min/week demonstrated the lowest AF risk with a J-shaped correlation between the extent of physical activity and AF risk; (3) this J-shaped correlation was observed in those with new-onset AF and a DM duration < 5 years, and was slightly attenuated in those with a DM duration of 5–9 years; (4) in patients with new-onset DM and a DM duration < 5 years and of 5–9 years, those engaging in 1,000–1,499 MET-min/week exhibited the lowest AF risk; and (5) for patients with a DM duration ≥ 10 years, higher physical activity levels led to a significantly lower risk of AF, and a longer DM duration amplified the positive impact of physical activity on AF risk (**Graphical abstract**).

In this large-scale, nationwide, population-based study, we analyzed the effect of physical activity on the risk of incident AF according to the duration of DM. We demonstrated that even among patients with a longer DM duration, those who engaged in a substantial amount of physical activity showed a lower relative risk of AF than those with new-onset AF who remained physically inactive.

Exercise improves glycemic control and reduces HbA1c levels in patients with DM regardless of the type of exercise [26]. Concerning the intensity of physical activity, the current recommendations for both aerobic and resistance exercises suggest engaging in moderate-intensity workouts as a minimum, with the option to target a more vigorous intensity if desired [27]. Consequently, the most recent consensus from the American College of Sports Medicine highlights the importance of regular physical activity and encourages individuals with T2DM to reduce their sedentary time and break up sitting periods, in line with the 2018 Physical Activity Guidelines for Americans [20, 28]. Nonetheless, the impact of physical activity on the risk of cardiovascular disease has remained complex [29].

One concern regarding physical activity intensity and AF is that excessive endurance exercise might increase the risk of AF [30, 31]. In a large cohort of Swedish men, excessive exercise among young adults was associated with an increased risk of AF [32]. Additionally, a previous systematic review established that athletes have a higher risk of AF than nonathletes [11]. However, one pooled observational cohort study encompassing more than 2 million participants identified no general association between regular physical activity and AF risk in overall population [33]. Nonetheless, the study did note a disparity between sexes, showing an increased risk of AF in men and a reduced risk in women, with the quality of evidence ranging from low to moderate.

Previous research has also demonstrated a U-shaped correlation between physical activity and AF [34]. Concerning the amount of physical activity and AF in previous nationwide population studies, the presence of physical activity has consistently shown the possibility of a beneficial effect on AF risk reduction [8, 9, 12]. Considering the outcomes of this study in conjunction with prior research, the relationship between physical activity and AF appears to be complex, and is influenced by multiple factors including age, sex, and comorbidity-related elements such as DM duration. Furthermore, our findings support the favorable effects of physical activity without necessitating concerns about the heightened risks associated with excessive exercise during the different stages of DM.

In a previous nationwide population study that focused on cardiovascular diseases (MI and stroke), regular exercise was associated with a dose-dependent decrease in risk among patients with T2DM, particularly in individuals aged 65 years and older [35]. Concerning the outcome of AF, we previously demonstrated a comparable pattern of risk reduction, with a more pronounced effect observed in individuals aged ≥ 65 years [12]. In the previous study, subgroup analyses based on the duration of DM were conducted but only considered durations of < 5 years and > 5 years [12]. The present study contributes to the literature by examining the effects of physical activity on distinct durations of DM.

A longer DM duration is associated with elevated rates of microvascular complications, macrovascular complications, and mortality [15, 36]. Additionally, the progression of DM was associated with a 3% annual increase in the incidence of AF [37]. Within this study, among the physically inactive participants, the crude incidence of AF increased in tandem with DM duration. The increased incidence of AF in individuals with diabetes may be attributed to factors such as left atrial enlargement, interstitial fibrosis in the atrial myocardium, and electrical remodeling [38, 39]. Moreover, findings from an in vitro study found a potential link between advanced glycation end products and heightened atrial myocyte senescence, contributing to a susceptibility to AF [40].

As the risk of AF varies with different durations of DM, we explored the potential effect of physical activity on AF risk. Individuals engaging in 1,000–1,499 MET-min/week demonstrated the lowest risk of AF. When compared with physically inactive individuals with new-onset DM, a more substantial reduction in AF risk was evident among those with heightened physical activity, particularly those with an extended DM duration. This outcome contrasts with the findings from the nationwide cohort study conducted by Jin et al. [41] In their research, Jin and his colleagues found a U-shaped correlation between physical activity and AF risk, with no significant risk reduction in highly active individuals, akin to new onset DM in our study. [41] A potential explanation for the varying effects of physical activity on different DM durations could be changes in the autonomic nervous system. As DM duration increases, so does the prevalence of autonomic neuropathy [42, 43]. Physical activity, shown to beneficially affect heart rate variability in a previous study [44], may counteract this alteration and enhance the positive effect on AF risk in groups with longer DM duration.

In contrast to earlier investigations that demonstrated an augmented risk of AF associated with excessive physical activity [30, 31], our study did not identify an elevated AF risk in any of the analyzed groups. Only in the new-onset DM group did those engaging in physical activity exceeding 1,500 MET-min/week exhibit a risk profile comparable to that of the physically inactive cohort. Nevertheless, the favorable impact of regular physical activity was most prominent in individuals with a long DM duration or a heightened burden of comorbidities, as evidenced by the attenuated J-curve observed in the cubic spline curve. This underscores the salutary role of routine physical activity in mitigating the risk of AF, particularly in patients with substantial comorbidities.

Engaging in regular physical activity is uncommon in individuals with DM. For example, the absence of participation in seeking diet or exercise advice remained steady (< 30% of Americans) from 2005 to 2015 [45, 46]. In this study, approximately half (50.3%) of the patients diagnosed with T2DM participated in physical activity of at least moderate intensity every week. Nevertheless, the occurrence of weekly physical activity diminished with longer durations of DM. Furthermore, individuals who engaged in physical activity at least five times per week constituted approximately 20% of the entire population. In terms of age, individuals with DM aged between 30 and 49 years old were more prone to receiving diet or exercise counseling than those aged 75 years and above [45]. Given the low occurrence of physical activity and the limited participation of older individuals, greater emphasis should be placed on promoting exercise in patients with DM regardless of their DM duration, based on the positive effects of physical activity, as shown in this study.

### Study limitations

This study had several limitations. First, this was an observational retrospective study. Nonetheless, the strength of this study is the inclusion of over two million participants. Second, there is the potential for recall bias due to the methodology employed. The assessment of physical activity relied on self-reported questionnaires that captured patient behaviors from the preceding week. Third, physically inactive patients comprise a heterogeneous population, including individuals who are medically incapable of engaging in exercise and those who exhibit a lack of concern about adopting a healthy lifestyle. Fourth, only leisure-time activity was accounted for and not occupational or household activities. Fifth, this study did not perform multiple (potentially underpowered) subgroup analyses, which could have depicted some variations based on sex or age. Finally, owing to the nature of the study design, the precise mechanisms underlying reduced AF associated with increased physical activity could not be identified.

## Conclusions

In conclusion, among patients with DM, longer DM duration was associated with a higher risk of incident AF, whereas increased physical activity generally reduced the risk of AF. Engaging in > 1,500 MET-min/week was associated with the greatest reduction in AF risk in patients with a longer DM duration, highlighting the potential benefits of higher activity levels for AF prevention.

### Electronic supplementary material

Below is the link to the electronic supplementary material.

Supplementary Material 1

## Data Availability

Data supporting the findings of this study are available from the corresponding author upon reasonable request.

## Abbreviations

AF: atrial fibrillation
CI: confidence interval
CKD: chronic kidney disease
DM: diabetes mellitus
HR: hazard ratio
ICD-10-CM: International Classification of Disease, Tenth Revision of Clinical Modification
IR: incidence rate
MI: myocardial infarction
NHIS: Korean National Health Insurance Service
SD: standard deviation

## References

[1] Zheng Y, Ley SH, Hu FB (2018). Global aetiology and epidemiology of type 2 diabetes mellitus and its complications. Nat Rev Endocrinol.

[2] Davies MJ, Aroda VR, Collins BS, Gabbay RA, Green J, Maruthur NM, Rosas SE, Del Prato S, Mathieu C, Mingrone G (2022). Management of hyperglycaemia in type 2 diabetes, 2022. A consensus report by the American Diabetes Association (ADA) and the European Association for the Study of Diabetes (EASD). Diabetologia.

[3] Rowlands A, Davies M, Dempsey P, Edwardson C, Razieh C, Yates T (2021). Wrist-worn accelerometers: recommending ~ 1.0 mg as the minimum clinically important difference (MCID) in daily average acceleration for inactive adults. Br J Sports Med.

[4] Yates T, Haffner SM, Schulte PJ, Thomas L, Huffman KM, Bales CW, Califf RM, Holman RR, McMurray JJ, Bethel MA (2014). Association between change in daily ambulatory activity and cardiovascular events in people with impaired glucose tolerance (NAVIGATOR trial): a cohort analysis. Lancet.

[5] Geng T, Wang Y, Lu Q, Zhang YB, Chen JX, Zhou YF, Wan Z, Guo K, Yang K, Liu L (2022). Associations of New-Onset Atrial Fibrillation with risks of Cardiovascular Disease, chronic kidney Disease, and Mortality among patients with type 2 diabetes. Diabetes Care.

[6] Seyed Ahmadi S, Svensson AM, Pivodic A, Rosengren A, Lind M (2020). Risk of atrial fibrillation in persons with type 2 diabetes and the excess risk in relation to glycaemic control and renal function: a Swedish cohort study. Cardiovasc Diabetol.

[7] Schnabel RB, Yin X, Gona P, Larson MG, Beiser AS, McManus DD, Newton-Cheh C, Lubitz SA, Magnani JW, Ellinor PT (2015). 50 year trends in atrial fibrillation prevalence, incidence, risk factors, and mortality in the Framingham Heart Study: a cohort study. Lancet.

[8] Lee SR, Choi EK, Ahn HJ, Han KD, Oh S, Lip GYH (2020). Association between clustering of unhealthy lifestyle factors and risk of new-onset atrial fibrillation: a nationwide population-based study. Sci Rep.

[9] Park CS, Han KD, Choi EK, Kim DH, Lee HJ, Lee SR, Oh S (2021). Lifestyle is associated with atrial fibrillation development in patients with type 2 diabetes mellitus. Sci Rep.

[10] Drca N, Wolk A, Jensen-Urstad M, Larsson SC (2015). Physical activity is associated with a reduced risk of atrial fibrillation in middle-aged and elderly women. Heart.

[11] Abdulla J, Nielsen JR (2009). Is the risk of atrial fibrillation higher in athletes than in the general population? A systematic review and meta-analysis. Europace.

[12] Park CS, Choi EK, Kyung D, Yoo J, Ahn HJ, Kwon S, Lee SR, Oh S, Lip GYH (2023). Physical activity changes and the risk of Incident Atrial Fibrillation in patients with type 2 diabetes Mellitus: a Nationwide Longitudinal follow-up Cohort Study of 1.8 million subjects. Diabetes Care.

[13] Cheol Seong S, Kim YY, Khang YH, Heon Park J, Kang HJ, Lee H, Do CH, Song JS, Hyon Bang J, Ha S (2017). Data Resource Profile: the National Health Information Database of the National Health Insurance Service in South Korea. Int J Epidemiol.

[14] Lee W-C, Lee S-Y. National Health Screening Program of Korea. *jkma* 2010, 53(5):363–370.

[15] Zoungas S, Woodward M, Li Q, Cooper ME, Hamet P, Harrap S, Heller S, Marre M, Patel A, Poulter N (2014). Impact of age, age at diagnosis and duration of diabetes on the risk of macrovascular and microvascular complications and death in type 2 diabetes. Diabetologia.

[16] de Jong M, Woodward M, Peters SAE (2022). Duration of diabetes and the risk of major cardiovascular events in women and men: a prospective cohort study of UK Biobank participants. Diabetes Res Clin Pract.

[17] Ohkuma T, Peters SAE, Jun M, Harrap S, Cooper M, Hamet P, Poulter N, Chalmers J, Woodward M (2020). Sex-specific associations between cardiovascular risk factors and myocardial infarction in patients with type 2 diabetes: the ADVANCE-ON study. Diabetes Obes Metab.

[18] Chun MY (2012). Validity and reliability of Korean version of international physical activity questionnaire short form in the elderly. Korean J Fam Med.

[19] Fogelholm M, Malmberg J, Suni J, Santtila M, Kyröläinen H, Mäntysaari M, Oja P (2006). International Physical Activity Questionnaire: validity against fitness. Med Sci Sports Exerc.

[20] Piercy KL, Troiano RP, Ballard RM, Carlson SA, Fulton JE, Galuska DA, George SM, Olson RD (2018). The physical activity guidelines for americans. JAMA.

[21] Ahn HJ, Lee SR, Choi EK, Han KD, Jung JH, Lim JH, Yun JP, Kwon S, Oh S, Lip GYH (2021). Association between exercise habits and stroke, heart failure, and mortality in Korean patients with incident atrial fibrillation: a nationwide population-based cohort study. PLoS Med.

[22] Jeong SW, Kim SH, Kang SH, Kim HJ, Yoon CH, Youn TJ, Chae IH (2019). Mortality reduction with physical activity in patients with and without cardiovascular disease. Eur Heart J.

[23] Choi EK (2020). Cardiovascular Research using the Korean National Health Information Database. Korean Circ J.

[24] Bansal N, Zelnick LR, Alonso A, Benjamin EJ, de Boer IH, Deo R, Katz R, Kestenbaum B, Mathew J, Robinson-Cohen C (2017). eGFR and Albuminuria in relation to risk of Incident Atrial Fibrillation: a Meta-analysis of the Jackson Heart Study, the multi-ethnic study of atherosclerosis, and the Cardiovascular Health Study. Clin J Am Soc Nephrol.

[25] Lee SR, Choi EK, Han KD, Lee SH, Oh S (2020). Effect of the variability of blood pressure, glucose level, total cholesterol level, and body mass index on the risk of atrial fibrillation in a healthy population. Heart Rhythm.

[26] Snowling NJ, Hopkins WG (2006). Effects of different modes of exercise training on glucose control and risk factors for complications in type 2 diabetic patients: a meta-analysis. Diabetes Care.

[27] Colberg SR, Sigal RJ, Fernhall B, Regensteiner JG, Blissmer BJ, Rubin RR, Chasan-Taber L, Albright AL, Braun B (2010). Exercise and type 2 diabetes: the American College of Sports Medicine and the American Diabetes Association: joint position statement executive summary. Diabetes Care.

[28] Kanaley JA, Colberg SR, Corcoran MH, Malin SK, Rodriguez NR, Crespo CJ, Kirwan JP, Zierath JR (2022). Exercise/Physical activity in individuals with type 2 diabetes: a Consensus Statement from the American College of Sports Medicine. Med Sci Sports Exerc.

[29] Chudyk A, Petrella RJ (2011). Effects of exercise on cardiovascular risk factors in type 2 diabetes: a meta-analysis. Diabetes Care.

[30] Laukkanen JA, Kunutsor SK, Ozemek C, Mäkikallio T, Lee DC, Wisloff U, Lavie CJ (2019). Cross-country skiing and running’s association with cardiovascular events and all-cause mortality: a review of the evidence. Prog Cardiovasc Dis.

[31] Scheer V, Tiller NB, Doutreleau S, Khodaee M, Knechtle B, Pasternak A, Rojas-Valverde D (2022). Potential long-term health problems Associated with Ultra-endurance running: a narrative review. Sports Med.

[32] Drca N, Wolk A, Jensen-Urstad M, Larsson SC (2014). Atrial fibrillation is associated with different levels of physical activity levels at different ages in men. Heart.

[33] Kunutsor SK, Seidu S, Mäkikallio TH, Dey RS, Laukkanen JA (2021). Physical activity and risk of atrial fibrillation in the general population: meta-analysis of 23 cohort studies involving about 2 million participants. Eur J Epidemiol.

[34] Heitmann KA, Løchen ML, Stylidis M, Hopstock LA, Schirmer H, Morseth B. Associations between physical activity, left atrial size and incident atrial fibrillation: the Tromsø Study 1994–2016. Open Heart 2022, 9(1). 10.1136/openhrt-2021-001823 PMC8788327 35074937

[35] Jung I, Moon SJ, Kwon H, Park SE, Han K-D, Rhee E-J, Lee W-Y (2022). Effects of physical activity on cardiovascular outcomes and mortality in Korean patients with diabetes: a nationwide population-based cohort study. Cardiovasc Prev Pharmacotherapy.

[36] Rawshani A, Rawshani A, Franzén S, Sattar N, Eliasson B, Svensson AM, Zethelius B, Miftaraj M, McGuire DK, Rosengren A (2018). Risk factors, mortality, and Cardiovascular outcomes in patients with type 2 diabetes. N Engl J Med.

[37] Dublin S, Glazer NL, Smith NL, Psaty BM, Lumley T, Wiggins KL, Page RL, Heckbert SR (2010). Diabetes mellitus, glycemic control, and risk of atrial fibrillation. J Gen Intern Med.

[38] Fu L, Rao F, Lian F, Yang H, Kuang S, Wu S, Deng C, Xue Y (2019). Mechanism of electrical remodeling of atrial myocytes and its influence on susceptibility to atrial fibrillation in diabetic rats. Life Sci.

[39] Bohne LJ, Johnson D, Rose RA, Wilton SB, Gillis AM (2019). The Association between Diabetes Mellitus and Atrial Fibrillation: clinical and mechanistic insights. Front Physiol.

[40] Zheng DL, Wu QR, Zeng P, Li SM, Cai YJ, Chen SZ, Luo XS, Kuang SJ, Rao F, Lai YY (2022). Advanced glycation end products induce senescence of atrial myocytes and increase susceptibility of atrial fibrillation in diabetic mice. Aging Cell.

[41] Jin MN, Yang PS, Song C, Yu HT, Kim TH, Uhm JS, Sung JH, Pak HN, Lee MH, Joung B (2019). Physical activity and risk of Atrial Fibrillation: a Nationwide Cohort Study in General Population. Sci Rep.

[42] Zoppini G, Cacciatori V, Raimondo D, Gemma M, Trombetta M, Dauriz M, Brangani C, Pichiri I, Negri C, Stoico V (2015). Prevalence of Cardiovascular Autonomic Neuropathy in a cohort of patients with newly diagnosed type 2 diabetes: the Verona newly diagnosed type 2 diabetes study (VNDS). Diabetes Care.

[43] Vinik AI, Maser RE, Mitchell BD, Freeman R (2003). Diabetic autonomic neuropathy. Diabetes Care.

[44] Picard M, Tauveron I, Magdasy S, Benichou T, Bagheri R, Ugbolue UC, Navel V, Dutheil F (2021). Effect of exercise training on heart rate variability in type 2 diabetes mellitus patients: a systematic review and meta-analysis. PLoS ONE.

[45] Shah MK, Moore MA, Narayan KMV, Ali MK (2019). Trends in Lifestyle Counseling for adults with and without diabetes in the U.S., 2005–2015. Am J Prev Med.

[46] Morrato EH, Hill JO, Wyatt HR, Ghushchyan V, Sullivan PW (2007). Physical activity in U.S. adults with diabetes and at risk for developing diabetes, 2003. Diabetes Care.

