# Supplementary material for: Effect of physical activity on incident atrial fibrillation in individuals with varying duration of diabetes: a nationwide population study
Source: Cardiovasc Diabetol. 2024 Mar 30;23:115. doi: 10.1186/s12933-024-02194-2 (PMC10981812; doi:10.1186/s12933-024-02194-2)
Supplement: Supplementary file 1 — Supplementary Material 1 [file 12933_2024_2194_MOESM1_ESM.docx]

Supplementary Material

**Supplementary Tables**

**Table S1.** Definitions of covariates.

**Table S2**. Relationship between CV risk factors and AF risk among total study population.

**Table S3**. Relationship between CV risk factors and AF risk across DM duration groups.

**Table S4.** Baseline characteristics of the study population according to DM duration and MET/min–week.

**Table S5**. Risk of incident AF according to DM duration and physical activity.

**Supplementary Figure**

**Figure S1.** Cumulative incidence curves of AF stratified by physical activity in different diabetes mellitus duration groups: (A) total (B) New onset (C) diabetes mellitus duration <5 years (D) diabetes mellitus 5–9 years (D) diabetes mellitus duration ≥10 years.

**Supplementary Tables**

**Supplementary Table S1. Definitions of covariates.**

| **Diagnosis** | **ICD-10-CM code and definition** | **Diagnostic definition** |
| --- | --- | --- |
| **Inclusion/exclusion criteria** |  |  |
| **Atrial fibrillation** | I48.0-48.4–I48.9 | Admission ≥ 1 or outpatient department ≥ 2 |
| **Diabetes mellitus** | E11-E14; and minimum one prescription of anti-diabetic drugs (sulfonylurea–metformin–meglitinide–thiazolidinedione–dipeptidyl peptidase-4 inhibitor–α-glucosidase inhibitor–and insulin). | Admission≥1 or outpatient department≥1 |
|  | Or fasting glucose level ≥ 126 mg/dL | Index health examination |
| **Comorbidities** |  |  |
| **Chronic kidney disease** | Estimated glomerular filtration rate <60 ml/min/1.73m^2^ | Index health examination |
| **Dyslipidemia** | E78 | Admission or outpatient department≥1 |
|  | Or Total cholesterol ≥ 240 mg/dL | Index health examination |
| **Heart failure** | I50 | Admission or outpatient department≥1 |
| **Hypertension** | I10-I13–I15; and minimum one prescription of anti-hypertensive medication (thiazide–loop diuretic–aldosterone antagonist–alpha-/beta-blocker–calcium channel blocker–angiotensin-converting enzyme inhibitor–and angiotensin II receptor blocker). | Admission≥1 or outpatient department≥2 |
|  | or systolic/diastolic blood pressure ≥ 140/90 mmHg | Index health examination |
| **Prior MI** | I21–I22 | Admission or outpatient department≥1 |
| **Prior stroke** | I63–I64 | Admission or outpatient department≥1 |
| **Peripheral artery disease** | I70–I73 | Admission or outpatient department≥1 |
| **Health exam questionnaire** |  |  |
| **Smoking**  **Ex**  **Current** | Ex-smoker at the 1^st^ examination and sustaining non-smoking till the 2^nd^ examination.  Current smoker at the 2^nd^ examination regardless of the smoking status at the 1^st^ examination. | Index health examination |
| **Alcohol consumption**  **Mild to moderate**  **Heavy** | Alcohol consumption >0g to <30g per day  Alcohol consumption ≥30g per day | Index health examination |
| **Low income** | Income lowest 20% among the entire Korean population and supported by the medical aid | Index health examination |

Abbreviations: ICD–international classification of disease; CM–clinical modification.

**Table S2**. Relationship between CV risk factors and AF risk among total study population.

|  | **Overall** | | | |
| --- | --- | --- | --- | --- |
|  | **Univariable** | | **Multivariable** | |
|  | **HR (95% C.I)** | **P-value** | **HR (95% C.I)** | **P-value** |
| **Age per 1 year** | 1.070 (1.069, 1.071) | <.001 | 1.066 (1.065, 1.067) | <.001 |
| **Sex (Male)** | 1.065 (1.045, 1.085) | <.001 | 1.454 (1.419, 1.491) | <.001 |
| **BMI per 1 kg/m^2^** | 0.995 (0.992, 0.997) | <.001 | 1.029 (1.026, 1.032) | <.001 |
| **Fasting glucose per 1 mg/dL** | 0.998 (0.997, 0.998) | <.001 | 1.001 (1.000, 1.001) | <.001 |
| **Low Income** | 1.043 (1.020, 1.066) | <.001 | 1.104 (1.080, 1.129) | <.001 |
| **Smoking** |  | <.001 |  | <.001 |
| **Non** | 1 (Ref.) |  | 1 (Ref.) |  |
| **Ex** | 1.035 (1.013, 1.058) |  | 1.024 (0.997, 1.051) |  |
| **Current** | 0.725 (0.707, 0.743) |  | 1.121 (1.088, 1.155) |  |
| **Drinking** |  | <.001 |  | <.001 |
| **Non** | 1 (Ref.) |  | 1 (Ref.) |  |
| **Mild** | 0.689 (0.674, 0.703) |  | 1.000 (0.976, 1.024) |  |
| **Heavy** | 0.745 (0.720, 0.771) |  | 1.145 (1.103, 1.188) |  |
| **Hypertension** | 2.166 (2.122, 2.212) | <.001 | 1.270 (1.241, 1.300) | <.001 |
| **Dyslipidemia** | 1.071 (1.052, 1.091) | <.001 | 0.895 (0.878, 0.912) | <.001 |
| **CKD** | 2.597 (2.539, 2.656) | <.001 | 1.265 (1.234, 1.298) | <.001 |
| **Heart Failure** | 1.786 (1.740, 1.834) | <.001 | 1.296 (1.261, 1.332) | <.001 |
| **PAD** | 1.140 (1.117, 1.164) | <.001 | 0.984 (0.964, 1.005) | 0.136 |
| **Stroke** | 3.248 (3.144, 3.355) | <.001 | 1.787 (1.728, 1.848) | <.001 |
| **MI** | 1.646 (1.612, 1.680) | <.001 | 1.120 (1.097, 1.144) | <.001 |
| **Insulin** | 2.362 (2.312, 2.413) | <.001 | 1.274 (1.246, 1.303) | <.001 |
| **OHA≥3** | 2.207 (2.142, 2.273) | <.001 | 1.362 (1.321, 1.405) | <.001 |
| **Systolic BP, per 1 mmHg** | 1.010 (1.010, 1.011) | <.001 | 1.000 (1.000, 1.001) | 0.288 |
| **eGFR per 1 mL/min/1.73m^2^** | 0.987 (0.987, 0.987) | <.001 | 1.000 (1.000, 1.000) | 0.096 |
| **Proteinuria** | 1.915 (1.863, 1.969) | <.001 | 1.519 (1.476, 1.564) | <.001 |

**Table S3**. Relationship between CV risk factors and AF risk across DM duration groups.

|  | **New onset** | | | | | **<5 years** | | | | **5–9 years** | | | | | | | **≥10 years** | | |  | |
| --- | --- | --- | --- | --- | --- | --- | --- | --- | --- | --- | --- | --- | --- | --- | --- | --- | --- | --- | --- | --- | --- |
|  | **Univariable** | | | **Multivariable** | | **Univariable** | | **Multivariable** | | **Univariable** | | **Multivariable** | | | **Univariable** | | | | **Multivariable** | | |
|  | **HR (95% CI)** | **P-value** | **HR (95% CI)** | | **P-value** | **HR (95% CI)** | **P-value** | **HR (95% CI)** | **P-value** | **HR (95% CI)** | **P-value** | | **HR (95% CI)** | **P-value** | | **HR (95% CI)** | | **P-value** | | **HR (95% CI)** | **P-value** |
| **Age per 1 year** | 1.07 (1.07–1.07) | <.001 | 1.07 (1.07–1.07) | | <.001 | 1.07 (1.06–1.07) | <.001 | 1.06 (1.06–1.06) | <.001 | 1.06 (1.06–1.06) | <.001 | | 1.06 (1.06–1.06) | <.001 | | 1.06 (1.06–1.06) | | <.001 | | 1.05 (1.05–1.06) | <.001 |
| **Sex (Male)** | 1.09 (1.04–1.14) | <.001 | 1.52 (1.44–1.62) | | <.001 | 1.13 (1.09–1.17) | <.001 | 1.53 (1.46–1.61) | <.001 | 1.14 (1.10–1.19) | <.001 | | 1.45 (1.38–1.53) | <.001 | | 1.13 (1.10–1.17) | | <.001 | | 1.37 (1.32–1.43) | <.001 |
| **BMI per 1 kg/m^2^** | 0.98 (0.97–0.98) | <.001 | 1.03 (1.02–1.03) | | <.001 | 0.99 (0.98–0.99) | 0.006 | 1.02 (1.02–1.03) | <.001 | 1.01 (1.00–1.01) | <.001 | | 1.02 (1.02–1.03) | <.001 | | 1.02 (1.02–1.03) | | <.001 | | 1.03 (1.02–1.03) | <.001 |
| **Fasting glucose**  **per 1 mg/dL** | 0.99 (0.99–0.99) | <.001 | 1.00 (1.00–1.00) | | <.001 | 0.99 (0.99–0.99) | <.001 | 1.00 (0.99–1.00) | 0.079 | 0.99 (0.99–0.99) | <.001 | | 1.00 (1.00–1.00) | 0.178 | | 0.99 (0.99–0.99) | | <.001 | | 1.00 (1.00–1.00) | 0.0001 |
| **Low Income** | 1.12 (1.07–1.18) | <.001 | 1.08 (1.03–1.14) | | <.001 | 1.06 (1.01–1.10) | 0.009 | 1.12 (1.07–1.17) | <.001 | 1.03 (0.98–1.08) | 0.163 | | 1.11 (1.06–1.16) | <.001 | | 0.97 (0.94–1.01) | | 0.250 | | 1.08 (1.03–1.12) | <.001 |
| **Smoking** |  |  |  | |  |  |  |  |  |  |  | |  |  | |  | |  | |  |  |
| **Non** | 1 (Ref.) | <.001 | 1 (Ref.) | | 0.001 | 1 (Ref.) | <.001 | 1 (Ref.) | <.001 | 1 (Ref.) | <.001 | | 1 (Ref.) | 0.006 | | 1 (Ref.) | | <.001 | | 1 (Ref.) | <.001 |
| **Ex** | 1.10 (1.05–1.15) |  | 1.05 (0.99–1.11) | |  | 1.02 (0.97–1.07) |  | 0.96 (0.91–1.02) |  | 1.10 (1.05–1.15) |  | | 1.06 (1.00–1.12) |  | | 1.04 (1.00–1.08) | |  | | 1.01 (0.97–1.06) |  |
| **Current** | 0.71 (0.68–0.75) |  | 1.12 (1.05–1.19) | |  | 0.79 (0.75–0.83) |  | 1.09 (1.03–1.16) |  | 0.82 (0.78–0.87) |  | | 1.10 (1.03–1.18) |  | | 0.86 (0.82–0.90) | |  | | 1.14 (1.08–1.21) |  |
| **Drinking** |  |  |  | |  |  |  |  |  |  |  | |  |  | |  | |  | |  | 0.2687 |
| **Non** | 1 (Ref.) | <.001 | 1 (Ref.) | | <.001 | 1 (Ref.) | <.001 | 1 (Ref.) | 0.045 | 1 (Ref.) | <.001 | | 1 (Ref.) | 0.011 | | 1 (Ref.) | |  | | 1 (Ref.) |  |
| **Mild** | 0.70 (0.67–0.73) |  | 1.06 (1.01–1.12) | |  | 0.73 (0.70–0.76) |  | 0.98 (0.93–1.03) |  | 0.79 (0.75–0.82) |  | | 1.00 (0.95–1.06) |  | | 0.79 (0.76–0.82) | |  | | 0.97 (0.93–1.01) |  |
| **Heavy** | 0.88 (0.82–0.93) |  | 1.33 (1.24–1.43) | |  | 0.78 (0.73–0.84) |  | 1.08 (1.00–1.17) |  | 0.86 (0.79–0.92) |  | | 1.13 (1.04–1.23) |  | | 0.80 (0.74–0.86) | |  | | 1.03 (0.95–1.11) |  |
| **Hypertension** | 2.20 (2.11–2.29) | <.001 | 1.29 (1.23–1.36) | | <.001 | 2.04 (1.95–2.13) | <.001 | 1.30 (1.24–1.36) | <.001 | 1.88 (1.80–1.97) | <.001 | | 1.25 (1.19–1.31) | <.001 | | 1.86 (1.79–1.94) | | <.001 | | 1.21 (1.16–1.27) | <.001 |
| **Dyslipidemia** | 1.15 (1.10–1.20) | <.001 | 0.93 (0.89–0.97) | | 0.001 | 0.90 (0.87–0.94) | <.001 | 0.87 (0.84–0.91) | <.001 | 0.86 (0.82–0.89) | <.001 | | 0.86 (0.82–0.89) | <.001 | | 0.95 (0.92–0.98) | | 0.004 | | 0.90 (0.87–0.93) | <.001 |
| **CKD** | 3.14 (2.96–3.34) | <.001 | 1.25 (1.17–1.34) | | <.001 | 2.39 (2.27–2.52) | <.001 | 1.20 (1.13–1.27) | <.001 | 2.12 (2.02–2.23) | <.001 | | 1.23 (1.16–1.30) | <.001 | | 2.12 (2.05–2.19) | | <.001 | | 1.30 (1.24–1.35) | <.001 |
| **Heart Failure** | 4.27 (3.91–4.68) | <.001 | 1.89 (1.72–2.07) | | <.001 | 3.20 (3.00–3.42) | <.001 | 1.81 (1.69–1.93) | <.001 | 2.88 (2.68–3.08) | <.001 | | 1.82 (1.69–1.95) | <.001 | | 2.60 (2.47–2.74) | | <.001 | | 1.72 (1.63–1.81) | <.001 |
| **PAD** | 2.22 (2.10–2.35) | <.001 | 1.16 (1.10–1.23) | | <.001 | 1.62 (1.55–1.69) | <.001 | 1.14 (1.09–1.20) | <.001 | 1.39 (1.33–1.45) | <.001 | | 1.13 (1.08–1.18) | <.001 | | 1.24 (1.20–1.28) | | <.001 | | 1.07 (1.04–1.11) | <.001 |
| **Stroke** | 3.08 (2.91–3.26) | <.001 | 1.26 (1.19–1.34) | | <.001 | 2.36 (2.25–2.47) | <.001 | 1.31 (1.25–1.38) | <.001 | 2.01 (1.92–2.10) | <.001 | | 1.30 (1.24–1.36) | <.001 | | 1.81 (1.75–1.87) | | <.001 | | 1.24 (1.19–1.28) | <.001 |
| **MI** | 2.59 (2.39–2.81) | <.001 | 1.37 (1.26–1.49) | | <.001 | 2.14 (2.00–2.28) | <.001 | 1.37 (1.28–1.47) | <.001 | 1.87 (1.76–1.99) | <.001 | | 1.35 (1.27–1.44) | <.001 | | 1.84 (1.76–1.92) | | <.001 | | 1.35 (1.28–1.41) | <.001 |
| **Insulin** | - | - | - | | - | 1.53 (1.44–1.63) | <.001 | 1.36 (1.28–1.45) | <.001 | 1.48 (1.39–1.57) | <.001 | | 1.42 (1.34–1.51) | <.001 | | 1.37 (1.32–1.42) | | <.001 | | 1.21 (1.16–1.25) | <.001 |
| **OHA≥3** | - | - | - | | - | 0.84 (0.79–0.88) | <.001 | 1.04 (0.98–1.09) | 0.120 | 0.82 (0.79–0.86) | <.001 | | 0.97 (0.93–1.02) | 0.315 | | 0.82 (0.80–0.85) | | <.001 | | 0.94 (0.91–0.97) | <.001 |
| **Systolic BP, per 1 mmHg** | 1.00 (1.00–1.01) | <.001 | 0.99 (0.99–0.99) | | 0.004 | 1.01 (1.01–1.01) | <.001 | 1.00 (0.99–1.00) | 0.949 | 1.01 (1.01–1.01) | <.001 | | 1.00 (0.99–1.00) | 0.573 | | 1.01 (1.01–1.01) | | <.001 | | 1.00 (1.00–1.00) | 0.001 |
| **eGFR per 1 mL/min/1.73m^2^** | 0.99 (0.99–0.99) | <.001 | 1.00 (1.00–1.00) | | 0.185 | 0.98 (0.98–0.99) | <.001 | 1.00 (0.99–1.00) | 0.068 | 0.99 (0.99–0.99) | <.001 | | 1.00 (0.99–1.00) | 0.691 | | 0.98 (0.98–0.98) | | <.001 | | 0.99 (0.99–1.00) | 0.024 |
| **Proteinuria** | 1.81 (1.68–1.94) | <.001 | 1.56 (1.45–1.67) | | <.001 | 1.49 (1.39–1.59) | <.001 | 1.39 (1.30–1.49) | <.001 | 1.58 (1.48–1.69) | <.001 | | 1.38 (1.29–1.48) | <.001 | | 1.93 (1.86–2.01) | | <.001 | | 1.59 (1.53–1.66) | <.001 |

**Table S4.** Baseline characteristics of the study population according to DM duration and MET/min-week

(A) New onset

|  | **MET/min-week** | | | | | ***p*-value** |
| --- | --- | --- | --- | --- | --- | --- |
|  | **0 (n=** **151,684)** | **<500**  **(n=** **199,056)** | **500–999**  **(n=195,121)** | **1000–1499**  **(n=88,335)** | **≥ 1500**  **(n=70,536)** |  |
| **Age–years** |  |  |  |  |  |  |
| **Mean ± SD** | 55.5 **±** 13.0 | 52.4 **±** 12.1 | 52.7 **±** 12.1 | 52.7 **±** 11.5 | 55.2 **±** 11.7 | <.001 |
| **<40** | 9.7 | 13.3 | 13.3 | 12.5 | 9.2 |  |
| **40–64** | 67.9 | 71.5 | 70.3 | 72.6 | 69.5 |  |
| **≥65** | 22.4 | 15.2 | 16.3 | 15.0 | 21.3 |  |
| **Sex (women)** | 36.8 | 32.0 | 28.2 | 25.9 | 25.0 |  |
| **Comorbidities** |  |  |  |  |  |  |
| **Hypertension** | 45.7 | 42.4 | 43.5 | 43.4 | 46.4 | <.001 |
| **Dyslipidemia** | 35.9 | 35.8 | 35.0 | 34.4 | 34.4 | <.001 |
| **CKD** | 5.9 | 4.2 | 4.0 | 3.8 | 4.4 | <.001 |
| **Proteinuria** | 5.6 | 5.4 | 5.2 | 4.9 | 4.6 | <.001 |
| **Heart failure** | 1.9 | 1.3 | 1.2 | 1.1 | 1.3 | <.001 |
| **Prior MI** | 7.7 | 5.1 | 4.9 | 4.6 | 5.9 | <.001 |
| **Prior stroke** | 3.2 | 2.5 | 2.5 | 2.6 | 2.9 | <.001 |
| **PAD** | 9.6 | 7.2 | 7.4 | 6.8 | 8.2 | <.001 |
| **Social history** |  |  |  |  |  |  |
| **Smoking** |  |  |  |  |  | <.001 |
| **Non-smoker** | 53.3 | 46.3 | 43.7 | 42.8 | 44.9 |  |
| **Ex-smoker** | 15.6 | 21.7 | 24.2 | 27.8 | 29.0 |  |
| **Current smoker** | 31.2 | 32.0 | 32.1 | 29.4 | 26.0 |  |
| **Alcohol consumption** |  |  |  |  |  | <.001 |
| **Non-drinker** | 52.1 | 41.8 | 38.5 | 36.5 | 40.1 |  |
| **Mild to moderate (0–30 g/day)** | 33.7 | 45.3 | 48.0 | 50.9 | 46.3 |  |
| **Heavy (≥30g/day)** | 14.2 | 13.0 | 13.5 | 12.7 | 13.7 |  |
| **Regular physical activity 1^a^** | 0.0 | 44.0 | 71.2 | 100.0 | 100.0 | <.001 |
| **Regular physical activity 2^b^** | 0.0 | 1.3 | 7.0 | 64.8 | 99.3 | <.001 |
| **METs (min/week)** |  | 308.0 **±** 126.9 | 718.2 **±** 132.5 | 1211.7 **±** 123.7 | 1995.2 **±** 417.3 | <.001 |
| **Low income** | 23.7 | 20.3 | 20.0 | 18.8 | 20.0 | <.001 |
| **Medication** |  |  |  |  |  |  |
| **OHA ≥ 3** | - | | | | | |
| **Insulin usage** | - | | | | | |
| **DM duration (years)** | - | | | | | |
| **Health examination** |  |  |  |  |  |  |
| **BMI (kg/m^2^)** | 25.5 **±** 3.8 | 25.8 **±** 3.8 | 25.7 **±** 3.7 | 25.6 **±** 3.6 | 25.3 **±** 3.4 | <.001 |
| **Obesity** | 52.9 | 56.1 | 55.0 | 53.9 | 51.5 | <.001 |
| **WC (cm)** | 85.9 **±** 9.5 | 86.5 **±** 9.4 | 86.3 **±** 9.2 | 85.9 **±** 9.0 | 85.3 **±** 8.6 | <.001 |
| **Abdominal Obesity** | 40.4 | 40.9 | 39.1 | 36.1 | 33.3 | <.001 |
| **SBP (mmHg)** | 129.3 **±** 15.6 | 129.2 **±** 15.2 | 129.6 **±** 15.2 | 129.7 **±** 15.1 | 129.9 **±** 15.1 | <.001 |
| **DBP (mmHg)** | 80.0 **±** 10.4 | 80.5 **±** 10.3 | 80.5 **±** 10.3 | 80.5 **±** 10.2 | 80.1 **±** 10.1 | <.001 |
| **Laboratory results** |  |  |  |  |  |  |
| **eGFR (mL/min/1.73 m2)** | 93.0 **±** 58.4 | 94.1 **±** 60.7 | 94.2 **±** 59.1 | 94.1 **±** 63.0 | 92.8 **±** 60.6 | <.001 |
| **Fasting Glucose (mg/dL)** | 152.2 **±** 39.6 | 152.9 **±** 40.7 | 151.9 **±** 39.7 | 150.3 **±** 37.6 | 148.5 **±** 35.1 | <.001 |
| **Total cholesterol (mg/dL)** | 206.7 **±** 42.9 | 209.1 **±** 42.0 | 208.0 **±** 41.6 | 207.0 **±** 41.2 | 204.5 **±** 40.9 | <.001 |
| **HDL–C (mg/dL)** | 52.5 **±** 16.5 | 52.0 **±** 16.4 | 52.7 **±** 15.8 | 53.3 **±** 16.9 | 54.1 **±** 16.3 | <.001 |
| **LDL–C (mg/dL)** | 119.2 **±** 39.2 | 121.0 **±** 38.4 | 119.8 **±** 38.1 | 119.3 **±** 37.7 | 117.8 **±** 37.7 | <.001 |
| **TG (mg/dL)^c^** | 156.2 (155.8–156.7) | 162.2 (161.8–162.7) | 158.2 (157.7–158.6) | 153.5 (152.9–154.1) | 143.3 (142.7–143.9) | <.001 |

Categorical variables were presented as a percentage and continuous variables were presented as mean and standard deviation.

^a^Performing a moderate physical activity more than 30 minutes at least 5 times per week or strenuous physical activity more than 20 minutes at least 3 times per week.

^b^Performing a moderate physical activity more than 30 minutes at least 1 time per week or strenuous physical activity more than 20 minutes at least 1 time per week.

^c^TG was presented as geometric mean (95% confidence interval).

Abbreviation: BMI, body mass index; CKD, chronic kidney disease; DBP, diastolic blood pressure; DM, diabetes mellitus; eGFR, estimated glomerular filtration rate; HDL-C, high density lipoprotein-cholesterol; LDL-C, low density lipoprotein-cholesterol; MI, myocardial infarction; OHA, oral antihyperglycemic agent; PAD, peripheral artery disease; SBP, systolic blood pressure; TG, triglyceride; WC, waist circumference.

(B) DM duration < 5 years

|  | **MET/min–week** | | | | | ***p*-value** |
| --- | --- | --- | --- | --- | --- | --- |
|  | **0 (n=150,864)** | **<500**  **(n=180,403)** | **500–999**  **(n=175,075)** | **1000–1499**  **(n=75,923)** | **≥ 1500**  **(n=67,546)** |  |
| **Age–years** |  |  |  |  |  |  |
| **Mean ± SD** | 60.1 **±** 11.3 | 57.7 **±** 11.4 | 58.1 **±** 11.3 | 57.2 **±** 10.8 | 59.2 **±** 10.6 | <.001 |
| **<40** | 2.8 | 4.6 | 4.6 | 4.7 | 3.4 |  |
| **40–64** | 64.1 | 68.7 | 67.0 | 70.5 | 65.3 |  |
| **≥65** | 33.2 | 26.7 | 28.4 | 24.8 | 31.3 |  |
| **Sex (women)** | 48.7 | 44.5 | 40.6 | 37.1 | 36.3 |  |
| **Comorbidities** |  |  |  |  |  |  |
| **Hypertension** | 61.5 | 57.9 | 58.2 | 56.6 | 58.1 | <.001 |
| **Dyslipidemia** | 66.0 | 66.4 | 65.5 | 65.4 | 64.1 | <.001 |
| **CKD** | 7.9 | 6.3 | 6.0 | 5.5 | 6.2 | <.001 |
| **Proteinuria** | 5.9 | 5.7 | 5.5 | 5.1 | 4.7 | <.001 |
| **Heart failure** | 4.0 | 3.1 | 2.9 | 2.6 | 2.7 | <.001 |
| **Prior MI** | 12.3 | 9.7 | 9.7 | 8.6 | 10.0 | <.001 |
| **Prior stroke** | 5.2 | 4.4 | 4.4 | 4.3 | 4.6 | <.001 |
| **PAD** | 19.1 | 16.2 | 16.3 | 15.0 | 16.3 | <.001 |
| **Social history** |  |  |  |  |  |  |
| **Smoking** |  |  |  |  |  | <.001 |
| **Non-smoker** | 61.5 | 55.8 | 53.1 | 51.0 | 52.9 |  |
| **Ex-smoker** | 15.6 | 21.1 | 23.9 | 27.5 | 28.0 |  |
| **Current smoker** | 22.9 | 23.2 | 23.0 | 21.4 | 19.1 |  |
| **Alcohol consumption** |  |  |  |  |  | <.001 |
| **Non-drinker** | 67.2 | 58.9 | 56.5 | 53.3 | 57.3 |  |
| **Mild to moderate (0–30 g/day)** | 23.6 | 32.8 | 34.7 | 38.2 | 34.0 |  |
| **Heavy (≥30g/day)** | 9.2 | 8.3 | 8.8 | 8.5 | 8.7 |  |
| **Regular physical activity 1^a^** | 0.0 | 37.2 | 64.0 | 100.0 | 100.0 | <.001 |
| **Regular physical activity 2^b^** | 0.0 | 1.4 | 7.8 | 69.3 | 99.5 | <.001 |
| **METs (min/week)** | - | 304.6 **±** 125.1 | 708.5 **±** 130.6 | 1212.6 **±** 123.3 | 2017.3 **±** 429.3 | <.001 |
| **Low income** | 24.3 | 21.6 | 21.6 | 20.4 | 20.4 | <.001 |
| **Medication** |  |  |  |  |  |  |
| **OHA ≥ 3** | 19.3 | 19.1 | 18.3 | 17.4 | 16.4 | <.001 |
| **Insulin usage** | 7.7 | 6.6 | 6.6 | 6.1 | 6.7 | <.001 |
| **DM duration (years)** | 2.0 **±** 1.6 | 2.0 **±** 1.6 | 2.0 **±** 1.6 | 2.0 **±** 1.6 | 2.1 **±** 1.6 | <.001 |
| **Health examination** |  |  |  |  |  |  |
| **BMI (kg/m^2^)** | 25.9 **±** 3.7 | 25.9 **±** 3.7 | 25.7 **±** 3.6 | 25.7 **±** 3.5 | 25.4 **±** 3.4 | <.001 |
| **Obesity** | 57.5 | 57.6 | 55.8 | 55.4 | 52.5 | <.001 |
| **WC (cm)** | 87.1 **±** 9.3 | 87.1 **±** 9.2 | 86.7 **±** 9.0 | 86.4 **±** 8.9 | 85.9 **±** 8.7 | <.001 |
| **Abdominal Obesity** | 48.0 | 46.2 | 43.8 | 40.9 | 38.7 | <.001 |
| **SBP (mmHg)** | 128.2 **±** 15.1 | 127.5 **±** 14.6 | 127.7 **±** 14.5 | 127.4 **±** 14.3 | 127.7 **±** 14.4 | <.001 |
| **DBP (mmHg)** | 78.4 **±** 9.8 | 78.5 **±** 9.7 | 78.4 **±** 9.6 | 78.3 **±** 9.6 | 78.0 **±** 9.5 | <.001 |
| **Laboratory results** |  |  |  |  |  |  |
| **eGFR (mL/min/1.73 m2)** | 90.6 **±** 46.5 | 92.0 **±** 52.0 | 92.2 **±** 51.7 | 92.6 **±** 54.2 | 91.9 **±** 55.7 | <.001 |
| **Fasting Glucose (mg/dL)** | 139.3 **±** 49.6 | 140.0 **±** 47.5 | 138.0 **±** 45.8 | 136.7 **±** 43.4 | 133.3 **±** 41.3 | <.001 |
| **Total cholesterol (mg/dL)** | 186.4 **±** 44.3 | 185.8 **±** 43.7 | 184.3 **±** 42.8 | 182.9 **±** 42.3 | 181.0 **±** 41.8 | <.001 |
| **HDL-C (mg/dL)** | 50.5 **±** 14.4 | 50.2 **±** 13.6 | 50.8 **±** 14.5 | 51.0 **±** 13.5 | 51.6 **±** 14.8 | <.001 |
| **LDL-C (mg/dL)** | 103.8 **±** 39.2 | 103.4 **±** 38.8 | 102.2 **±** 38.1 | 101.3 **±** 37.9 | 100.5 **±** 36.9 | <.001 |
| **TG (mg/dL)^c^** | 142.9 (142.5–143.3) | 143.9 (143.5–144.3) | 139.0 (138.6–139.3) | 135.6 (135.0–136.1) | 127.5 (127.0–128.1) | <.001 |

Categorical variables were presented as a percentage and continuous variables were presented as mean and standard deviation.

^a^Performing a moderate physical activity more than 30 minutes at least 5 times per week or strenuous physical activity more than 20 minutes at least 3 times per week.

^b^Performing a moderate physical activity more than 30 minutes at least 1 time per week or strenuous physical activity more than 20 minutes at least 1 time per week.

^c^TG was presented as geometric mean (95% confidence interval).

Abbreviation: CKD, chronic kidney disease; DBP, diastolic blood pressure; DM, diabetes mellitus; eGFR, estimated glomerular filtration rate; MI, myocardial infarction; OHA, oral antihyperglycemic agent; PAD, peripheral artery disease; SBP, systolic blood pressure; TG, triglyceride; WC, waist circumference.

(C) DM duration 5–10 years

|  | **MET/min-week** | | | | | ***p*-value** |
| --- | --- | --- | --- | --- | --- | --- |
|  | **0 (n=107,692)** | **<500**  **(n=124,166)** | **500–999**  **(n=125,148)** | **1000–1499**  **(n=54,732)** | **≥ 1500**  **(n=53,132)** |  |
| **Age–years** |  |  |  |  |  |  |
| **Mean ± SD** | 63.5 **±** 10.6 | 61.2 **±** 10.7 | 61.5 **±** 10.6 | 60.3 **±** 10.0 | 62.1 **±** 9.7 | <.001 |
| **<40** | 0.8 | 1.6 | 1.6 | 1.8 | 1.1 |  |
| **40–64** | 55.1 | 61.6 | 60.1 | 64.8 | 58.3 |  |
| **≥65** | 44.1 | 36.8 | 38.3 | 33.5 | 40.6 |  |
| **Sex (women)** | 51.0 | 46.0 | 41.4 | 36.1 | 34.4 |  |
| **Comorbidities** |  |  |  |  |  |  |
| **Hypertension** | 68.3 | 64.5 | 64.2 | 62.1 | 63.7 | <.001 |
| **Dyslipidemia** | 65.1 | 66.1 | 65.0 | 64.6 | 63.4 | <.001 |
| **CKD** | 12.5 | 10.1 | 9.6 | 8.2 | 9.3 | <.001 |
| **Proteinuria** | 6.9 | 6.9 | 6.5 | 6.0 | 5.8 | <.001 |
| **Heart failure** | 4.3 | 3.3 | 3.1 | 2.8 | 2.8 | <.001 |
| **Prior MI** | 17.7 | 14.4 | 13.7 | 11.6 | 13.6 | <.001 |
| **Prior stroke** | 7.0 | 6.0 | 5.9 | 5.6 | 6.0 | <.001 |
| **PAD** | 24.0 | 21.5 | 21.2 | 19.7 | 20.7 | <.001 |
| **Social history** |  |  |  |  |  |  |
| **Smoking** |  |  |  |  |  | <.001 |
| **Non-smoker** | 64.6 | 58.4 | 55.6 | 52.1 | 53.4 |  |
| **Ex-smoker** | 15.3 | 20.8 | 24.1 | 28.7 | 29.5 |  |
| **Current smoker** | 20.1 | 20.8 | 20.4 | 19.2 | 17.1 |  |
| **Alcohol consumption** |  |  |  |  |  | <.001 |
| **Non-drinker** | 71.5 | 63.2 | 60.4 | 55.5 | 58.5 |  |
| **Mild to moderate (0–30 g/day)** | 20.8 | 29.7 | 32.0 | 36.5 | 33.3 |  |
| **Heavy (≥30g/day)** | 7.8 | 7.2 | 7.6 | 8.0 | 8.2 |  |
| **Regular physical activity 1** | 0.0 | 34.6 | 60.6 | 100.0 | 100.0 | <.001 |
| **Regular physical activity 2** | 0.0 | 1.6 | 8.1 | 70.9 | 99.6 | <.001 |
| **METs (min/week)** | - | 305.2 **±** 123.9 | 704.6 **±** 129.8 | 1213.3 **±** 123.7 | 2033.9 **±** 436.8 | <.001 |
| **Low income** | 25.1 | 22.8 | 22.2 | 21.0 | 21.3 | <.001 |
| **Medication** |  |  |  |  |  |  |
| **OHA ≥ 3** | 36.8 | 36.9 | 34.9 | 33.8 | 31.0 | <.001 |
| **Insulin usage** | 9.6 | 8.3 | 7.9 | 7.2 | 7.3 | <.001 |
| **DM duration (years)** | 7.4 **±** 1.5 | 7.4 **±** 1.5 | 7.4 **±** 1.5 | 7.4 **±** 1.5 | 7.5 **±** 1.5 | <.001 |
| **Health examination** |  |  |  |  |  |  |
| **BMI (kg/m^2^)** | 25.4 **±** 3.6 | 25.4 **±** 3.5 | 25.2 **±** 3.3 | 25.2 **±** 3.2 | 25.0 **±** 3.1 | <.001 |
| **Obesity** | 52.6 | 52.2 | 49.8 | 49.2 | 47.0 | <.001 |
| **WC (cm)** | 86.7 **±** 9.1 | 86.5 **±** 8.9 | 86.1 **±** 8.7 | 85.8 **±** 8.6 | 85.4 **±** 8.4 | <.001 |
| **Abdominal Obesity** | 46.6 | 44.0 | 41.1 | 37.8 | 35.9 | <.001 |
| **SBP (mmHg)** | 128.5 **±** 15.3 | 127.7 **±** 14.7 | 127.9 **±** 14.6 | 127.7 **±** 14.3 | 128.1 **±** 14.5 | <.001 |
| **DBP (mmHg)** | 77.4 **±** 9.6 | 77.4 **±** 9.5 | 77.3 **±** 9.4 | 77.3 **±** 9.3 | 77.1 **±** 9.3 | <.001 |
| **Laboratory results** |  |  |  |  |  |  |
| **eGFR (mL/min/1.73 m2)** | 86.7 **±** 43.5 | 88.8 **±** 51.4 | 89.0 **±** 49.9 | 90.2 **±** 54.6 | 88.8 **±** 51.8 | <.001 |
| **Fasting Glucose (mg/dL)** | 140.9 **±** 47.9 | 142.4 **±** 46.3 | 140.5 **±** 44.6 | 140.2 **±** 4 | 137.3 **±** 41.0 | <.001 |
| **Total cholesterol (mg/dL)** | 176.2 **±** 40.4 | 175.1 **±** 39.6 | 174.2 **±** 39.0 | 173.9 **±** 38.4 | 173.0 **±** 38.3 | <.001 |
| **HDL-C (mg/dL)** | 49.9 **±** 13.4 | 50.0 **±** 13.2 | 50.6 **±** 14.1 | 50.9 **±** 13.5 | 51.4 **±** 14.6 | <.001 |
| **LDL-C (mg/dL)** | 96.0 **±** 36.1 | 95.0 **±** 35.2 | 94.4 **±** 34.7 | 94.3 **±** 34.2 | 94.2 **±** 34.0 | <.001 |
| ***TG (mg/dL)** | 134.7 (134.3–135.1) | 134.4 (134.0–134.8) | 129.6 (129.2–130.0) | 126.6 (126.0–127.2) | 120.4 (119.8–120.9) | <.001 |

Categorical variables were presented as a percentage and continuous variables were presented as mean and standard deviation.
*TG was presented as geometric mean (95% confidence interval).
Abbreviation:CKD–chronic kidney disease; DBP–diastolic blood pressure; DM–diabetes mellitus; eGFR–estimated glomerular filtration rate; MI–myocardial infarction; OHA–oral antihyperglycemic agent; PAD–peripheral artery disease; SBP–systolic blood pressure; TG–triglyceride; WC–waist circumference.

(D) DM duration ≥10 years

|  | **MET/min-week** | | | | | ***p*-value** |
| --- | --- | --- | --- | --- | --- | --- |
|  | **0 (n=134,687)** | **<500**  **(n=146,728)** | **500–999**  **(n=153,155)** | **1000–1499**  **(n=65,785)** | **≥ 1500**  **(n=72,718)** |  |
| **Age–years** |  |  |  |  |  |  |
| **Mean ± SD** | 66.8±10.0 | 64.8±10.1 | 65.1±9.7 | 63.6±9.4 | 65.1±8.9 | <.001 |
| **<40** | 0.3 | 0.6 | 0.6 | 0.7 | 0.4 |  |
| **40–64** | 42.0 | 48.6 | 46.8 | 52.8 | 46.6 |  |
| **≥65** | 57.7 | 50.9 | 52.7 | 46.5 | 53.1 |  |
| **Sex (women)** | 53.6 | 48.5 | 43.0 | 36.1 | 33.8 |  |
| **Comorbidities** |  |  |  |  |  |  |
| **Hypertension** | 71.8 | 68.5 | 67.9 | 65.6 | 66.7 | <.001 |
| **Dyslipidemia** | 64.8 | 66.1 | 64.3 | 63.9 | 62.8 | <.001 |
| **CKD** | 22.1 | 18.6 | 17.4 | 14.7 | 15.3 | <.001 |
| **Proteinuria** | 11.9 | 11.1 | 10.5 | 10.2 | 9.4 | <.001 |
| **Heart failure** | 5.7 | 4.6 | 4.1 | 3.6 | 3.7 | <.001 |
| **Prior MI** | 24.8 | 20.3 | 19.4 | 16.7 | 18.4 | <.001 |
| **Prior stroke** | 9.2 | 8.1 | 7.8 | 7.1 | 7.5 | <.001 |
| **PAD** | 29.0 | 27.3 | 26.6 | 25.0 | 25.9 | <.001 |
| **Social history** |  |  |  |  |  |  |
| **Smoking** |  |  |  |  |  | <.001 |
| **Non-smoker** | 69.0 | 63.2 | 60.0 | 54.5 | 56.2 |  |
| **Ex-smoker** | 14.6 | 20.4 | 23.8 | 29.7 | 29.9 |  |
| **Current smoker** | 16.5 | 16.4 | 16.1 | 15.8 | 13.9 |  |
| **Alcohol consumption** |  |  |  |  |  | <.001 |
| **Non-drinker** | 77.0 | 70.2 | 67.2 | 60.7 | 63.4 |  |
| **Mild to moderate (0–30 g/day)** | 17.1 | 24.5 | 27.1 | 33.1 | 30.1 |  |
| **Heavy (≥30g/day)** | 6.0 | 5.4 | 5.8 | 6.2 | 6.5 |  |
| **Regular physical activity 1^a^** | 0.0 | 30.8 | 55.6 | 100.0 | 100.0 | <.001 |
| **Regular physical activity 2^b^** | 0.0 | 1.6 | 8.6 | 72.9 | 99.7 | <.001 |
| **METs (min/week)** | - | 305.8 **±** 122.4 | 698.6 **±** 127.7 | 1213.7 **±** 124.3 | 2057.0 **±** 445.0 | <.001 |
| **Low income** | 22.5 | 21.5 | 20.9 | 20.3 | 19.8 | <.001 |
| **Medication** |  |  |  |  |  |  |
| **OHA ≥ 3** | 52.6 | 53.0 | 51.7 | 50.8 | 48.6 | <.001 |
| **Insulin usage** | 22.8 | 21.2 | 19.8 | 18.5 | 18.7 | <.001 |
| **DM duration (years)** | 12.7±1.4 | 12.6±1.4 | 12.7±1.3 | 12.6±1.3 | 12.7±1.3 | <.001 |
| **Health examination** |  |  |  |  |  |  |
| **BMI (kg/m^2^)** | 24.7±3.5 | 24.7±3.3 | 24.5±3.2 | 24.4±3.0 | 24.3±3.0 | <.001 |
| **Obesity** | 44.3 | 43.1 | 40.5 | 40.0 | 37.6 | <.001 |
| **WC (cm)** | 85.9±9.1 | 85.5±8.7 | 85.1±8.4 | 84.8±8.3 | 84.6±8.2 | <.001 |
| **Abdominal Obesity** | 43.4 | 40.0 | 36.9 | 33.7 | 31.9 | <.001 |
| **SBP (mmHg)** | 129.1±15.9 | 128.5±15.4 | 128.5±15.2 | 128.0±14.8 | 128.5±15.0 | <.001 |
| **DBP (mmHg)** | 75.9±9.8 | 75.8±9.6 | 75.7±9.5 | 75.7±9.3 | 75.5±9.4 | <.001 |
| **Laboratory results** |  |  |  |  |  |  |
| **eGFR (mL/min/1.73 m2)** | 80.4±44.0 | 82.6±47.2 | 83.4±48.5 | 84.8±48.0 | 84.4±49.8 | <.001 |
| **Fasting Glucose (mg/dL)** | 149.0±55.2 | 149.4±52.5 | 147.3±50.3 | 146.9±48.3 | 144.0±46.0 | <.001 |
| **Total cholesterol (mg/dL)** | 171.31±40.2 | 170.3±39.3 | 169.4±38.5 | 168.6±37.7 | 167.9±37.1 | <.001 |
| **HDL-C (mg/dL)** | 49.2±13.9 | 49.5±13.7 | 50.2±13.7 | 50.5±14.1 | 51.2±15.0 | <.001 |
| **LDL-C (mg/dL)** | 92.9±35.4 | 92.2±34.6 | 91.7±33.9 | 91.2±33.7 | 91.2±32.9 | <.001 |
| **TG (mg/dL)^c^** | 129.4 (129.1–129.8) | 126.4 (126.0–126.7) | 121.6 (121.2–121.9) | 118.0 (117.6–118.5) | 112.1 (111.7–112.6) | <.001 |

Categorical variables were presented as a percentage and continuous variables were presented as mean and standard deviation.

^a^Performing a moderate physical activity more than 30 minutes at least 5 times per week or strenuous physical activity more than 20 minutes at least 3 times per week.

^b^Performing a moderate physical activity more than 30 minutes at least 1 time per week or strenuous physical activity more than 20 minutes at least 1 time per week.

^c^TG was presented as geometric mean (95% confidence interval).
Abbreviation: CKD, chronic kidney disease; DBP, diastolic blood pressure; DM, diabetes mellitus; eGFR, estimated glomerular filtration rate; MI, myocardial infarction; OHA, oral antihyperglycemic agent; PAD, peripheral artery disease; SBP, systolic blood pressure; TG, triglyceride; WC, waist circumference.

**Table S5.** (A) Risk of incident AF according to DM duration and physical activity in Model 1

| **DM Duration** | **METs–Min/week** | **Model 1 Composite** | ***p*-value** | **Model 1 Subgroup** | ***p*-value** | ***p*-for-interaction** |
| --- | --- | --- | --- | --- | --- | --- |
| **Total** | **0** | 1 (Reference) | <.001 |  |  |  |
|  | **<500** | 0.77 (0.75–0.78) |  |  |  |  |
|  | **500–999** | 0.78 (0.76–0.80) |  |  |  |  |
|  | **1000–1499** | 0.68 (0.65–0.70) |  |  |  |  |
|  | **≥1500** | 0.82 (0.79–0.85) |  |  |  |  |
| **New onset** | **0** | 1 (Reference) | <.001 | 1 (Reference) | 0.001 | 0.002 |
|  | **<500** | 0.71 (0.67–0.75) |  | 0.71 (0.67–0.75) |  |  |
|  | **500–999** | 0.77 (0.73–0.81) |  | 0.77 (0.73–0.81) |  |  |
|  | **1000–1499** | 0.69 (0.65–0.75) |  | 0.69 (0.65–0.75) |  |  |
|  | **≥1500** | 0.95 (0.89–1.02) |  | 0.95 (0.89–1.02) |  |  |
| **<5 years** | **0** | 1.23 (1.16–1.29) |  | 1 (Reference) | 0.009 |  |
|  | **<500** | 0.96 (0.91–1.01) |  | 0.78 (0.74–0.82) |  |  |
|  | **500–999** | 0.98 (0.93–1.04) |  | 0.80 (0.76–0.84) |  |  |
|  | **1000–1499** | 0.89 (0.85–0.95) |  | 0.72 (0.68–0.77) |  |  |
|  | **≥1500** | 1.01 (0.94–1.08) |  | 0.82 (0.77–0.88) |  |  |
| **5–9 years** | **0** | 1.58 (1.50–1.67) |  | 1 (Reference) | <.001 |  |
|  | **<500** | 1.27 (1.20–1.34) |  | 0.81 (0.76–0.85) |  |  |
|  | **500–999** | 1.28 (1.21–1.35) |  | 0.81 (0.77–0.86) |  |  |
|  | **1000–1499** | 1.02 (0.95–1.10) |  | 0.65 (0.60–0.70) |  |  |
|  | **≥1500** | 1.23 (1.14–1.30) |  | 0.78 (0.72–0.83) |  |  |
| **≥10 years** | **0** | 2.10 (2.00–2.21) |  | 1 (Reference) | <.001 |  |
|  | **<500** | 1.69 (1.60–1.77) |  | 0.80 (0.77–0.84) |  |  |
|  | **500–999** | 1.64 (1.56–1.72) |  | 0.78 (0.75–0.81) |  |  |
|  | **1000–1499** | 1.42 (1.33–1.51) |  | 0.67 (0.64–0.71) |  |  |
|  | **≥1500** | 1.54 (1.45–1.60) |  | 0.73 (0.69–0.77) |  |  |

Model 1: unadjusted

Abbreviation: AF, atrial fibrillation; DM, diabetes mellitus; IR, incidence rate; PY, person-year.

(B) Risk of incident AF according to DM duration and physical activity in Model 2

| **DM Duration** | **METs–Min/week** | **Model 2 Composite** | ***p*–value** | **Model 2 Subgroup** | ***p*–value** | ***p*–for–interaction** |
| --- | --- | --- | --- | --- | --- | --- |
| **Total** | **0** | 1 (Reference) | <.001 |  |  |  |
|  | **<500** | 0.89 (0.87–0.92) |  |  |  |  |
|  | **500–999** | 0.87 (0.85–090) |  |  |  |  |
|  | **1000–1499** | 0.81 (0.78–0.83) |  |  |  |  |
|  | **≥1500** | 0.83 (0.81–0.86) |  |  |  |  |
| **New onset** | **0** | 1 (Reference) | <.001 | 1 (Reference) | <.001 | <.001 |
|  | **<500** | 0.88 (0.83–0.93) |  | 0.88 (0.83–0.93) |  |  |
|  | **500–999** | 0.91 (0.86–0.96) |  | 0.91 (0.86–0.96) |  |  |
|  | **1000–1499** | 0.84 (0.78–0.91) |  | 0.84 (0.78–0.91) |  |  |
|  | **≥1500** | 0.97 (0.90–1.04) |  | 0.97 (0.90–1.04) |  |  |
| **<5 years** | **0** | 1.03 (0.97–1.08) |  | 1 (Reference) | <.001 |  |
|  | **<500** | 0.92 (0.87–0.97) |  | 0.90 (0.85–0.95) |  |  |
|  | **500–999** | 0.91 (0.86–0.96) |  | 0.88 (0.84–0.93) |  |  |
|  | **1000–1499** | 0.87 (0.81–0.93) |  | 0.85 (0.79–0.91) |  |  |
|  | **≥1500** | 0.87 (0.81–0.93) |  | 0.84 (0.79–0.90) |  |  |
| **5**–**9 years** | **0** | 1.10 (1.05–1.17) |  | 1 (Reference) | <.001 |  |
|  | **<500** | 1.00 (0.95–1.06) |  | 0.91 (0.86–0.96) |  |  |
|  | **500–999** | 0.98 (0.93–1.03) |  | 0.89 (0.84–0.93) |  |  |
|  | **1000–1499** | 0.84 (0.78–0.90) |  | 0.76 (0.71–0.82) |  |  |
|  | **≥1500** | 0.89 (0.83–0.96) |  | 0.81 (0.75–0.86) |  |  |
| **≥10 years** | **0** | 1.23 (1.17–1.30) |  | 1 (Reference) | <.001 |  |
|  | **<500** | 1.09 (1.04–1.14) |  | 0.88 (0.85–0.92) |  |  |
|  | **500–999** | 1.02 (0.97–1.08) |  | 0.83 (0.80–0.87) |  |  |
|  | **1000–1499** | 0.95 (0.90–1.02) |  | 0.77 (0.73–0.82) |  |  |
|  | **≥1500** | 0.94 (0.88–0.99) |  | 0.76 (0.72–0.80) |  |  |

Model 2: adjusted for age and sex

Abbreviation: AF, atrial fibrillation; DM, diabetes mellitus; IR, incidence rate; PY, person-year.

(C) Risk of incident AF according to DM duration and physical activity in Model 3

| **DM Duration** | **METs–Min/week** | **Model 3 Composite** | ***p*-value** | **Model 3 Subgroup** | ***p*-value** | ***p*-for-interaction** |
| --- | --- | --- | --- | --- | --- | --- |
| **Total** | **0** | 1 (Reference) | <.001 |  |  |  |
|  | **<500** | 0.91 (0.89–0.94) |  |  |  |  |
|  | **500–999** | 0.90 (0.88–0.93) |  |  |  |  |
|  | **1000–1499** | 0.85 (0.82–0.88) |  |  |  |  |
|  | **≥1500** | 0.88 (0.85–0.91) |  |  |  |  |
| **New onset** | **0** | 1 (Reference) | <.001 | 1 (Reference) | <.001 | <.001 |
|  | **<500** | 0.89 (0.84–0.94) |  | 0.89 (0.84–0.94) |  |  |
|  | **500–999** | 0.92 (0.87–0.98) |  | 0.92 (0.87–0.98) |  |  |
|  | **1000–1499** | 0.86 (0.80–0.93) |  | 0.86 (0.80–0.93) |  |  |
|  | **≥1500** | 0.99 (0.92–1.07) |  | 0.99 (0.92–1.07) |  |  |
| **<5 years** | **0** | 0.97 (0.92–1.02) |  | 1 (Reference) | <.001 |  |
|  | **<500** | 0.89 (0.85–0.94) |  | 0.92 (0.87–0.97) |  |  |
|  | **500–999** | 0.88 (0.84–0.93) |  | 0.91 (0.87–0.96) |  |  |
|  | **1000–1499** | 0.86 (0.80–0.92) |  | 0.89 (0.83–0.95) |  |  |
|  | **≥1500** | 0.86 (0.80–0.92) |  | 0.88 (0.83–0.95) |  |  |
| **5–9 years** | **0** | 1.02 (0.96–1.08) |  | 1 (Reference) | <.001 |  |
|  | **<500** | 0.95 (0.90–1.01) |  | 0.93 (0.89–0.99) |  |  |
|  | **500–999** | 0.94 (0.89–0.99) |  | 0.92 (0.88–0.97) |  |  |
|  | **1000–1499** | 0.82 (0.76–0.88) |  | 0.80 (0.75–0.87) |  |  |
|  | **≥1500** | 0.87 (0.81–0.94) |  | 0.85 (0.80–0.92) |  |  |
| **≥10 years** | **0** | 1.07 (1.01–1.12) |  | 1 (Reference) | <.001 |  |
|  | **<500** | 0.97 (0.92–1.02) |  | 0.91 (0.87–0.95) |  |  |
|  | **500–999** | 0.93 (0.89–0.98) |  | 0.87 (0.84–0.91) |  |  |
|  | **1000–1499** | 0.89 (0.83–0.95) |  | 0.83 (0.78–0.88) |  |  |
|  | **≥1500** | 0.87 (0.82–0.93) |  | 0.82 (0.78–0.87) |  |  |

Model 3: adjusted for age, sex, comorbidities (hypertension, dyslipidemia, chronic kidney disease), anti-hyperglycemic agent usage (more than three drug prescription, insulin), BMI, fasting glucose, low income, and social behavior (smoking, alcohol)

Abbreviation: AF, atrial fibrillation; DM, diabetes mellitus; IR, incidence rate; PY, person-year.

(D) Risk of incident AF according to DM duration and physical activity in Model 4

| **DM Duration** | **METs–Min/week** | **Model 4 Composite** | ***p*-value** | **Model 4 Subgroup** | ***p*-value** | ***p*-for-interaction** |
| --- | --- | --- | --- | --- | --- | --- |
| **Total** | **0** | 1 (Reference) | <.001 |  |  |  |
|  | **<500** | 0.92 (0.90–0.95) |  |  |  |  |
|  | **500–999** | 0.92 (0.90–0.94) |  |  |  |  |
|  | **1000–1499** | 0.87 (0.84–0.90) |  |  |  |  |
|  | **≥1500** | 0.90 (0.87–0.93) |  |  |  |  |
| **New onset** | **0** | 1 (Reference) | <.001 | 1 (Reference) | <.0001 | 0.002 |
|  | **<500** | 0.89 (0.84–0.95) |  | 0.89 (0.84–0.95) |  |  |
|  | **500–999** | 0.93 (0.88–0.99) |  | 0.93 (0.88–0.99) |  |  |
|  | **1000–1499** | 0.87 (0.81–0.94) |  | 0.87 (0.81–0.94) |  |  |
|  | **≥1500** | 1.01 (0.94–1.08) |  | 1.01 (0.94–1.08) |  |  |
| **<5 years** | **0** | 0.96 (0.91–1.02) |  | 1 (Reference) | 0.001 |  |
|  | **<500** | 0.90 (0.85–0.95) |  | 0.93 (0.89–0.98) |  |  |
|  | **500–999** | 0.89 (0.84–0.94) |  | 0.93 (0.88–0.98) |  |  |
|  | **1000–1499** | 0.87 (0.81–0.94) |  | 0.91 (0.85–0.97) |  |  |
|  | **≥1500** | 0.87 (0.81–0.93) |  | 0.90 (0.85–0.97) |  |  |
| **5–9 years** | **0** | 1.00 (0.95–1.06) |  | 1 (Reference) | 0.008 |  |
|  | **<500** | 0.95 (0.90–1.01) |  | 0.95 (0.90–0.99) |  |  |
|  | **500–999** | 0.94 (0.89–0.99) |  | 0.94 (0.89–0.99) |  |  |
|  | **1000–1499** | 0.83 (0.77–0.89) |  | 0.82 (0.77–0.88) |  |  |
|  | **≥1500** | 0.88 (0.82–0.94) |  | 0.88 (0.82–0.94) |  |  |
| **≥10 years** | **0** | 1.04 (0.98–1.09) |  | 1 (Reference) | <.001 |  |
|  | **<500** | 0.96 (0.91–1.01) |  | 0.92 (0.88–0.96) |  |  |
|  | **500–999** | 0.93 (0.88–0.98) |  | 0.89 (0.86–0.93) |  |  |
|  | **1000–1499** | 0.89 (0.83–0.95) |  | 0.86 (0.81–0.91) |  |  |
|  | **≥1500** | 0.87 (0.82–0.93) |  | 0.85 (0.80–0.89) |  |  |

Model 4: adjusted for age, sex, comorbidities (hypertension, dyslipidemia, chronic kidney disease), anti-hyperglycemic agent usage (more than three drug prescription, insulin), BMI, fasting glucose, low income, social behavior (smoking, alcohol), heart failure, prior myocardial infarction, prior stroke, and peripheral artery disease.

Abbreviation: AF, atrial fibrillation; DM, diabetes mellitus; IR, incidence rate; PY, person-year.

**Figure S1.** Cumulative incidence curves of AF stratified by physical activity in different diabetes mellitus duration groups: (A) total (B) New onset (C) diabetes mellitus duration <5 years (D) diabetes mellitus 5–9 years (D) diabetes mellitus duration ≥10 years.


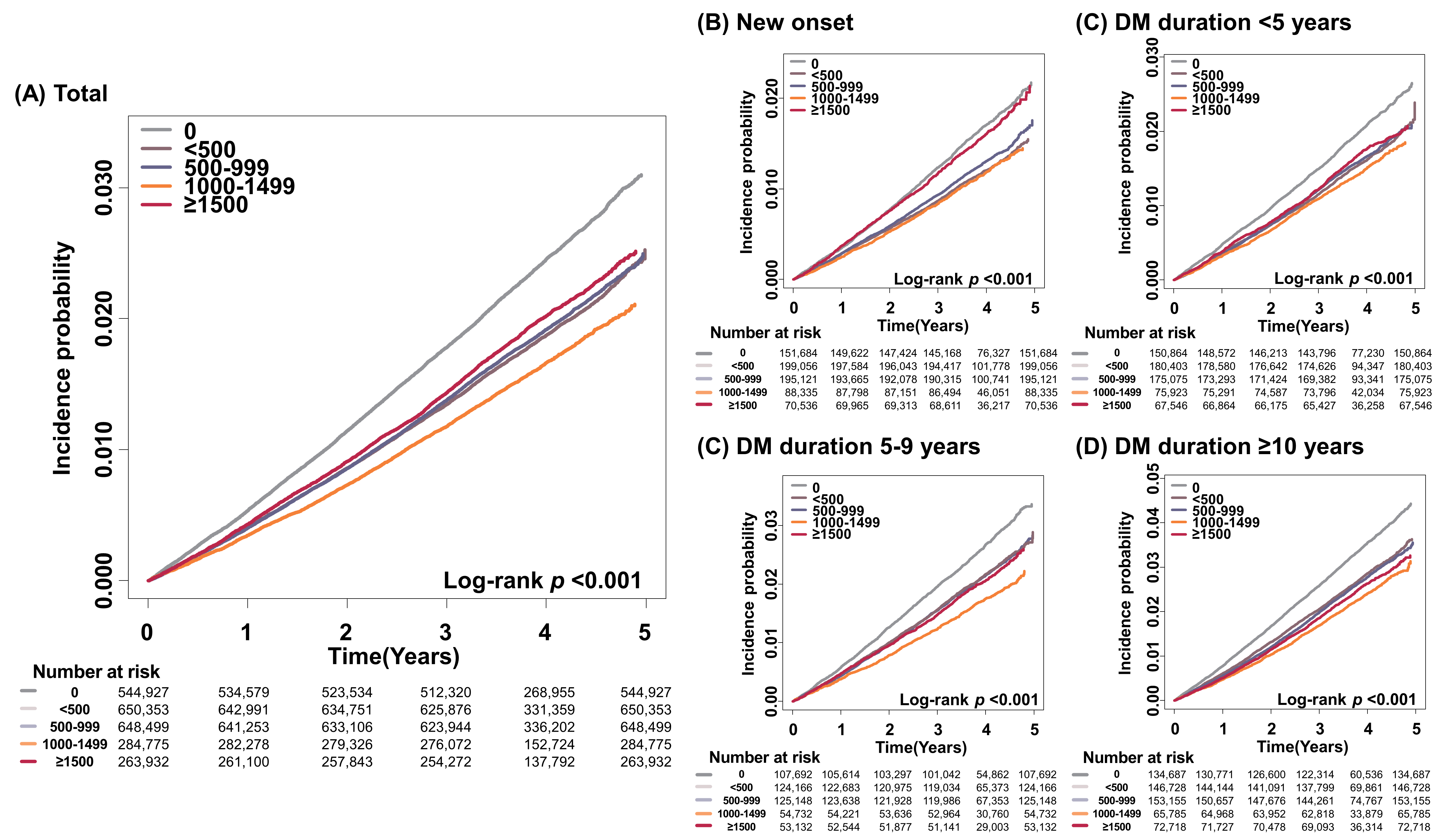


Abbreviation: DM–diabetes mellitus.
